# Relationship between the provision of procedures in psychosocial care centers and socioeconomic factors: an ecological study^*^

**DOI:** 10.1590/1518-8345.8045.4979

**Published:** 2026-11-13

**Authors:** Anna Helen Silva Lima, Ardigleusa Alves Coelho, Gabriela Maria Cavalcanti Costa, Ricardo Alves de Olinda, Valdecir Carneiro da Silva

**Affiliations:** 1 Universidade Estadual da Paraíba, Departamento de Enfermagem, Campina Grande, PB, Brazil.; 2 Scholarship holder at the Conselho Nacional de Desenvolvimento Científico e Tecnológico (CNPq), Brazil.; 3 Universidade Estadual da Paraíba, Departamento de Estatística, Campina Grande, PB, Brazil.

**Keywords:** Mental Health, Psychosocial Care Center, Community Mental Health Services, Access to Healthcare, Health Information Systems, Health Management.

## Abstract

**Objective:**

to analyze the correlation between the supply of procedures performed in the Psychosocial Care Centers and the socioeconomic indicators in municipalities with populations above 100 thousand inhabitants.

**Method:**

this is an ecological study covering 312 municipalities and 1,127 services. In the modeling, data were extracted from the historical series of outpatient production of the Health Information System and socioeconomic indicators from the Atlas of Human Development.

**Results:**

only 12 capitals recorded harm reduction actions; 93% of the records are more frequent in the capitals. A positive correlation was observed between home care and per capita income; and a negative correlation between psychosocial rehabilitation and illiteracy rate. It is noteworthy that the Municipal Human Development Index and *per capita* income were correlated with most of the procedures, while the illiteracy rate correlated with 64.7% of them. Statistically significant differences were found between medium and large municipalities.

**Conclusion:**

the relationship between the procedures recorded in the Psychosocial Care Centers and the socioeconomic indicators may indicate the influence of multifactorial issues on mental health care.

## Highlights

**(1)** Mental Health care in Brazil is influenced by social inequalities.

**(2)** Care in Community Mental Health Centers is concentrated in large centers.

**(3)** Failure to notify records of procedures in the Information Systems.

**(4)** CAPS procedures are mainly correlated with the Municipal Human Development Index (IDHM) and income.

**(5)** The supply of CAPS procedures is related to socioeconomic factors.

## Introduction

Mental disorders (MD) are a global public health problem and are among the main non-fatal causes of early death, contributing to the reduction of life expectancy by about 20 years compared to the general population^1^. However, only 12% of affected people have access to health services in low-income countries, according to the World Report on Mental Health^2^ ^-^ ^3^. In Brazil, funding for mental health is insufficient to meet care needs, representing only 1.6% of total resources in 2016^3^. The strengthening of community mental health services is foreseen in the Comprehensive Mental Health Action Plan (2013 - 2030), which proposes the increase of facilities in 80% of the countries as a strategy to reduce psychiatric rehospitalization rates^2^ ^-^ ^5^.

The Brazilian psychiatric reform restructured mental health care, ranging from the regulation of rights to the provision of community-based care^3^. In this context, Psychosocial Care Centers (CAPS), regulated by the Ministry of Health Ordinances No. 224/1992^6^ and No. 336/2002^7^, constitute territorial reference services, of an open nature, aimed at promoting the autonomy and social (re)integration of users^8^. Between 1998 and 2020, the number of CAPS increased from 148 to 2,657 CAPS^9^, consolidating itself as components of the Strategic Action of the Psychosocial Care Network (RAPS) and as substitute devices for the asylum model, ensuring the transit of users through services of various complexities^1^ ^,^ ^10^. From 2016 onwards, however, the national political scenario began to negatively impact the operationalization of the CAPS, due to the adoption of neoliberal and conservative strategies that reduced funding and destructured the National Mental Health Policy (PNSM) through the dissemination of normative documents and the publication of technical notes^4^ ^,^ ^11^.

The activities performed at the CAPS are recorded according to the standardization of procedures based on the Table of the Management System of Procedures, Medications, Orthoses, Prostheses and Special Materials of the Unified Health System (SIGTAP), recorded using instruments such as the Registry of Outpatient Health Actions - Psychosocial (RAAS - Psychosocial), Consolidated Outpatient Production Bulletin (BPA-C) and Individualized Outpatient Production Bulletin (BPA-I). grouped in Health Information Systems^12^. The information provided is not used for financing purposes, since the financial transfer of the CAPS is fixed, depending on its typology^13^.

However, there is still no consensus about the objects of evaluation of mental health services in Brazil, due to the need to contemplate the subjectivities that involve the production of care^14^. In the CAPS, the evaluation instruments frequently used focus on user satisfaction and the perception of professionals through scales - Evaluation of Patient Satisfaction with Mental Health Services (SATIS-BR) and Evaluation of the Impact of Work in Mental Health Services (*Impacto*-BR)^15^. Thus, the evaluation and monitoring of the service acts as a crucial tool to ensure effective and efficient services, through the qualification of access and care, in addition to ensuring that the scenarios of negligence previously witnessed do not recur^16^ ^-^ ^17^.

Despite the influence of socioeconomic conditions on illness and care follow-up^18^, the analysis of the relationship between the procedures performed at the CAPS and the municipal socioeconomic indicators remains incipient in the scientific scenario. The evaluation of secondary data, historically used predominantly for administrative purposes of team organization, enables a more comprehensive understanding of the inequities that permeate the provision of care, as it allows the identification of care patterns in different territorial contexts. In the context of mental health, health data enable the monitoring and evaluation of services and public policies, considering regional socioeconomic specificities^13^.

Therefore, considering the population established for the installation of the different CAPS modalities, the study chose to evaluate medium and large municipalities, which are the main responsible for commuting in search of health services^19^. That said, the objective is to analyze the correlation between the supply of procedures performed in the CAPS and the socioeconomic indicators in municipalities with a population size above 100 thousand inhabitants, so that, thus, inferences can be produced about equity in access to community-based care services for people with mental and behavioral disorders in Brazil.

## Method

### Type of study

This is an ecological study design, based on the analysis of secondary data available in the Outpatient Information System of the Department of Informatics of the Unified Health System (SIA/SUS)^20^ ^)^ and in the Atlas of Human Development in Brazil, of the United Nations Development Program (PNUD)^21^. The study was conducted in accordance with the recommendations of the Strengthening the Reporting of Observational Studies in Epidemiology (STROBE).

### Research setting 

The study covered 312 Brazilian municipalities, selected based on population stratification - above 100 thousand inhabitants, according to data from the 2022 Census^22^. Including the 26 capitals, the Federal District and the other municipalities, distributed in the five regions: 27 municipalities in the North Region, 63 in the Northeast, 143 in the Southeast, 55 in the South and 24 in the Midwest. The selection of municipalities based on population stratification was due to a significant data gap. In this context, the outpatient production of a percentage of 1,127 CAPS (40%), regardless of their typology, out of a total of 2,836 (100%) existing in the country^22^ was analyzed. The geographic distribution of the CAPS included in the sample is as follows: 53 located in the North Region, 198 in the Northeast, 596 in the Southeast, 197 in the South and 83 in the Midwest.

### Population and sample

The sample consisted of 23 procedures recorded in three instruments used in the CAPS care routines, namely: RAAS - Psychosocial, BPA-C and BPA-I. Considering 15 procedures registered in the RAAS - Psychosocial: night reception of patients in CAPS; third-shift reception of patients in CAPS; daytime reception of patients in CAPS; individual patient care in CAPS; group patient care in CAPS; family care in CAPS; home care for CAPS patients; body practices in CAPS; expressive and communicative practices in CAPS; attention to crisis situations; psychosocial rehabilitation actions; promotion of contractuality; monitoring of adults with suffering or mental disorders resulting from the use of crack, alcohol and other drugs - Adult Shelter Unit and Monitoring of the child and adolescent population with suffering or mental disorders resulting from the use of crack, alcohol and other drugs - Child and Adolescent Shelter Unit. While a total of seven procedures are registered in the BPA-C, of which: actions to articulate intra and intersectoral networks; strengthening the protagonism of CAPS users and their families; matrix support of primary care teams; harm reduction actions; follow-up of therapeutic residential service by CAPS; support for residential services of a transitory nature by CAPS; matrix support of teams from urgent and emergency care points and reference hospital services for care for people with suffering or mental disorders and with health needs resulting from the use of alcohol, crack and other drugs. Finally, the initial reception by CAPS, registered in the BPA-I.

The inclusion criterion was the existence of records present in at least 10% of the 312 municipalities analyzed, 5 procedures were eliminated: Actions of articulation of intra and intersectoral networks; strengthening the protagonism of CAPS users and their families; Matrix support for Primary Care teams; Monitoring of the child and adolescent population with suffering or mental disorders resulting from the use of crack, alcohol and other drugs - Child and Adolescent Reception Unit (UAI) and matrix support of teams from urgent and emergency care points and reference hospital services for care of people with suffering or mental disorders and with health needs resulting from alcohol use, crack and other drugs. The final sample consisted of 18 procedures. For the municipalities, the inclusion criterion observed was the existence of records of procedures in at least one year of the historical series and a population greater than 100 thousand.

### Procedures and instruments for data collection

Data collection was carried out during the period from November 2023 to August 2024, through two stages: (1) through the Outpatient Information System of the Department of Informatics of the Unified Health System^20^ (https://datasus.saude.gov.br); and (2) through the Atlas of Human Development in Brazil^21^ (https://www.atlasbrasil.org.br).

In stage 1, initially, secondary data obtained by the SIA/SUS were collected on the records of procedures (dependent variables) in CAPS authorized by the Ministry of Health that presented outpatient production using the RAAS, BPA-C and BPA-I instruments.

For stage 2, the independent variables were the socioeconomic indicators available in the Atlas of Human Development in Brazil: Municipal Human Development Index (MHDI), Gini index, Theil L index, unemployment rate (population aged 18 years and over), illiteracy rate (population aged 15 years and over), per capita income (minimum wage) and proportion of poor people.

### Data analysis procedures

Initially, a descriptive analysis of the data was carried out with calculations and comparisons of the average supply in the CAPS procedures, considering groups of municipalities with populations between 100,000 and 199,999 (medium size) and those with more than 200,000 inhabitants (large size). For the dependent variables, the simple arithmetic mean of the historical series (2016 to 2022) was calculated by municipality. The data regarding the supply of procedures were standardized for comparison between the groups of municipalities based on the number of populations, by calculating the average number of procedures in the historical series by the number of inhabitants and multiplying the result by 10,000 inhabitants.

After that, the free software Jamovi, version 2.3^23^, was used to apply the statistical tests. First, the Shapiro-Wilk test was performed, indicating that the data are non-parametric. Spearman’s statistical correlation test was performed to evaluate the supply of procedures performed at the CAPS and socioeconomic indicators. In addition, the non-parametric Kruskal-Wallis ANOVA was performed to compare the supply of procedures between the groups of municipalities according to size. Statistical significance was set at 5%, i.e., a value of p<0.05.

### Ethical aspects

According to the Resolution of the National Health Council No. 510/2012^24^, the submission of research is waived when using data in the public domain and open access.

## Results

The distribution of the procedures offered by municipalities was as follows: individual care (100%), family care (99.6%), group care (99.3%), day reception (99%), initial reception (98.7%), home care (97.7%), care in crisis situations (94.5%), expressive and communicative practices (94.2%), body practices (93.2%), psychosocial rehabilitation actions (92.9%), promotion of contractuality (86.8%), night reception (68.9%), third-shift reception (53.8%), patient follow-up in the Therapeutic Residential Service (28.2%), follow-up in the Therapeutic Residential Service (20.1%), harm reduction actions (15%) and follow-up of adults in an Adult Reception Unit (10%).

Table 1 shows the rate (per 10 thousand inhabitants) of CAPS procedures according to outpatient production in Brazilian capitals. Records of procedures with high rates stand out for capitals in the following regions: Midwest (Brasília and Campo Grande); North (Palmas and Rio Branco); Northeast (João Pessoa and Recife); Southeast (São Paulo and Vitória); and South (Curitiba and Porto Alegre).

**Table 1:** Rates (per 10 thousand inhabitants) of CAPS* procedures in Brazilian capitals, 2016 to 2022. Brazil, 2024

Region	Capitals	Procedures							
		Night reception	Third-shift reception	Patient follow-up in TRS^†^	Day reception	Individual care	Group care	Family care	Initial reception
Midwest	Brasília	1.02	0.43	-	18.21	102.2	33.72	16.13	16.62
	Campo Grande	68.24	0.03	1.44	76.82	508.44	142.78	74.34	27.98
	Cuiabá	-	-	-	4.79	95.78	55.46	39.55	20.62
	Goiânia	0.12	0.10	1.58	2.54	63.21	29.33	13.53	7.14
North	Boa Vista	6.95	16.12	-	-	61.70	39.31	5.65	25.52
	Belém	1.68	-	-	5.00	186.25	17.56	53.64	19.32
	Macapá	-	-	-	6.21	47.80	28.82	10.34	-
	Manaus	1.12	-	-	3.33	123.94	11.69	35.70	12.85
	Palmas	14.25	1.12	-	56.19	555.70	182.20	70.57	62.88
	Rio Branco	12.76	-	-	20.47	230.34	10.72	3.30	9.99
Northeast	Aracaju	18.79	0.17	15.80	74.63	153.33	66.10	63.67	9.55
	Fortaleza	0.11	0.55	0.22	16.92	78.90	11.51	2.13	24.40
	João Pessoa	11.92	11.98	-	302.87	101.04	65.84	7.17	2.92
	Maceió	7.81	2.22	0.01	97.06	169.31	141.31	39.29	14.37
	Natal	10.88	-	0.96	3.53	138.00	119.50	10.50	10.05
	Recife	24.61	8.18	20.25	75.77	285.35	199.50	84.83	30.16
	Salvador	0.41	0.01	0.47	74.21	113.84	41.04	21.98	6.08
	São Luís	0.88	0.01	-	109.33	115.68	57.30	30.47	2.69
	Teresina	26.15	-	2.07	63.90	275.90	145.82	116.50	6.86
Southeast	Belo Horizonte	34.17	15.23	11.90	256.55	193.48	11.95	1.09	4.11
	Rio de Janeiro	7.37	6.19	4.01	45.55	120.99	24.61	28.76	8.83
	São Paulo	26.41	6.10	1.56	116.68	327.15	147.52	70.46	33.95
	Vitória	38.03	0.13	-	226.32	296.81	86.11	45.49	36.23
South	Curitiba	87.79	2.52	1.43	81.14	661.59	311.37	131,73	50.66
	Florianópolis	0.02	0.03	-	10.67	172.57	196.33	16.70	32.33
	Porto Alegre	17.33	8.04	-	32.33	224.33	45.57	33.83	18.54
**Region**	**Capitals**	**Procedures**								
		**Home care**	**Body practices**	**Expressive practices**	**Care in crisis situations**	**Harm reduction actions**	Follow- up in the TRS^†^ by CAPS*	**Psycosocial rehabilitation actions**	**Promotion of contractuality**	**Follow-up of adults in an ARU^‡^**
Midwest	Brasília	0.76	2.89	398	6.64	0.01	0.04	9.15	1.82	-
	Campo Grande	1.25	13.16	28.53	80.25	-	-	3.82	3.50	1.11
	Cuiabá	5.08	0.41	20.15	0.86	0.12	-	1.35	1.25	-
	Goiânia	0.53	5.10	3.80	0.92	0.02	0.03	1.98	1.79	-
North	Belém	1.33	10.05	9.20	3.02	-	0.21	9.20	11.23	-
	Boa Vista	1.03	5.84	1.17	2.44	0.10	0.46	-	0.05	0.86
	Macapá	0.05	0.05	2.22	3.57	-	-	0.07	0.04	-
	Manaus	0.88	6.69	6.12	2.01	-	0.14	6.12	7.47	-
	Palmas	18.75	13.06	5.15	2.63	0.36	0.45	13.89	0.51	-
	Porto Velho	4.64	32.51	8.41	7.13	0.10	-	3.43	0.34	-
	Rio Branco	0.56	0.03	0.82	-	0.59	-	6.13	7.68	4.62
Northeast	Aracaju	9.72	10.71	36.19	41.22	-	-	38.11	7.80	1.36
	Fortaleza	0.66	0.20	0.14	0.28	-	0.04	0.59	0.04	0.16
	João Pessoa	1.40	1.92	25.89	4.70	-	26.70	3.97	0.06	-
	Maceió	4.55	13.03	15.94	4.23	-	0.01	4.86	1.00	-
	Natal	0.14	0.95	2.76	2.07	-	0.01	0.79	0.24	-
	Recife	11.13	6.13	17.35	22.20	0.12	0.01	8.74	26.45	1.85
	Salvador	2.09	10.81	30.78	1.89	-	-	6.42	4.32	-
	São Luís	11.61	20.13	25.54	37.17	-	-	19.02	0.30	-
	Teresina	7.78	10.45	35.61	58.18	-	-	7.68	-	-
Southeast	Belo Horizonte	12.18	4.46	3.61	5.75	0.01	-	0.88	5.02	-
	Rio de Janeiro	2.75	6.98	22.00	12.21	1.08	0.03	11.63	6.81	-
	São Paulo	14.65	29.84	85.10	14.00	-	-	49.28	24.52	0.38
	Vitória	3.12	3.38	112.34	8.49	-	-	19.45	10.83	-
South	Curitiba	4.75	28.03	44.43	68.98	-	0.02	80.13	36.37	-
	Florianópolis	0.06	4.47	16.26	5.53	-	-	3.23	6.27	-
	Porto Alegre	0.91	9.70	10.41	6.58	2.56	0.01	22.50	6.89	0.01

Source: SIA/SUS _^(20)^_ , 2016-2022. *CAPS = Psychosocial Care Center; ^†^TRS = Therapeutic Residential Service; ^‡^ARU = Adult Reception Unit

In this context, as shown in Table 2, elaborated based on the offer of procedures in non-medium-sized capitals, it is noteworthy that certain Federative Units do not have occurrences for non-capital municipalities in the sample, since the municipalities in the interior do not have the population size established in the survey.Table 3 presents the scenario of non-capitals with large municipalities. Occurrences with high rates were observed for the following regions and not state capitals: Central-West (Mato Grosso and Mato Grosso do Sul); North (Rondônia and Pará); Northeast (Maranhão and Paraíba); Southeast (Minas Gerais and São Paulo); and South (Paraná and Santa Catarina).

It is worth mentioning that procedures such as third-shift reception and night care are concentrated in this group, since they are restricted to services that operate uninterruptedly, 24 hours a day. Therefore, the lack of records of these procedures in non-medium-sized capital municipalities is an expected finding in the set of data analyzed.

**Table 2:** Average rates (per 10 thousand inhabitants) of CAPS* procedures in non-medium-sized capital cities, 2016 to 2022. Brazil, 2024

Region	Non-capitals	Procedures							
		Night reception	Third-shift reception	Patient follow-up in TRS^†^	Day reception	Individual care	Group care	Family care	Initial reception
Midwest	Goiás (Non-capital)	0.16	0.07	1.65	15.21	129.03	67.45	6.01	18.32
	Mato Grosso (Non-capital)	0.09	-	-	61.83	194.70	26.89	52.07	26.00
	Mato Grosso do Sul (Non-capital)	-	-	-	50.62	151.46	0.30	6.88	19.81
North	Amapá (Non-capital)	-	-	-	1.17	17.79	5.70	0.75	1.63
	Amazonas (Non-capital)	3.23	-	-	3.94	162.54	67.71	20.14	13.63
	Pará (Non-capital)	6.27	1.24	-	47.99	227.16	112.74	65.39	18.16
	Rondônia (Non-capital)	0.38	-	-	58.78	138.38	2.32	0.14	0.37
Northeast	Bahia (Non-capital)	0.45	0.50	-	154.89	267.26	120.32	83.49	29.63
	Ceará (Non-capital)	1.22	0.13	-	49.17	241.49	18.22	15.25	20.73
	Maranhão (Non-capital)	27.80	26.40	-	816.66	843.12	209.06	84.45	114.72
	Paraíba (Non-capital)	1.78	1.73	1.04	72.59	541.96	5678.58	718.45	22.35
	Pernambuco (Non-capital)	12.74	18.03	12.85	18.12	170.13	186.90	110.74	19.91
	Piauí (Non-capital)	-	0.98	-	0.22	28.47	4.31	0.39	0.10
	Rio Grande do Norte (Non-capital)	1.58	-	-	44.49	116.76	31.17	51.25	0.11
	Sergipe (Non-capital)	0.45	8.12	233.02	24.23	230.68	268.88	70.25	11.09
Southeast	Espírito Santo (Non-capital)	0.42	0.15	-	123.43	169.93	42.84	14.28	9.02
	Minas Gerais (Non-capital)	26.75	7.60	73.71	169.92	424.85	127.07	63.48	35.34
	Rio de Janeiro (Non-capital)	0.82	0.24	6.13	236.19	160.75	114.21	44.56	26.12
	São Paulo (Non-capital)	8.11	0.42	8.88	100.14	285.34	118.81	45.71	15.60
South	Paraná (Non-capital)	0.55	1.18	-	102.73	257.47	258.92	56.41	20.64
	Rio Grande do Sul (Non-capital)	6.62	0.47	2.55	32.79	338.96	105.49	56.34	28.92
	Santa Catarina (Non-capital)	0.81	27.28	-	52.52	309.46	178.26	49.80	35.74
**Region**	**Non-capitals**	**Procedures**								
		**Home care**	**Body practices**	**Expressive practices**	**Care in crisis situations**	**Harm reduction actions**	Follow- up in the TRS^†^ by CAPS*	**Psycosocial rehabilitation actions**	**Promotion of contractuality**	**Follow-up of adults in an ARU^‡^**
Midwest	Goiás (Non-capital)	7.07	10.56	21.94	1.85	2.53	0.20	1.30	1.42	-
	Mato Grosso (Non-capital)	7.65	14.15	37.48	5.55	2.31	0.21	30.98	13.83	-
	Mato Grosso do Sul (Non- capital)	1.85	-	39.09	4.82	-	-	0.90	1.32	-
North	Amapá (Non-capital)	-	4.01	4.74	3.15	-	-	9.62	2.03	-
	Amazonas (Non-capital)	2.22	0.95	7.15	5.91	1.20	-	15.21	0.51	-
	Pará (Non-capital)	3.13	42.92	47.54	7.97	-	-	24.67	3.20	-
	Rondônia (Non-capital)	10.44	-	-	-	-	-	17.48	2.70	-
Northeast	Maranhão (Non-capital)	1.93	30.87	22.60	47.81	-	0.60	84.58	0.09	-
	Bahia (Non-capital)	20.56	55.67	80.21	2.28	0.27	-	45.86	40.45	-
	Ceará (Non-capital)	6.14	1.53	0.11	2.06	-	0.08	0.28	-	-
	Paraíba (Non-capital)	7.77	706.94	25.23	3.13	0.10	-	2.13	0.99	-
	Pernambuco (Non-capital)	6.36	8.66	33.95	45.55	-	0.47	7.40	42.62	0.63
	Piauí (Non-capital)	0.09	0.06	-	-	-	-	0.31	0.06	-
	Rio Grande do Norte (Non- capital)	1.42	1.65	2.66	0.08	-	-	0.55	0.17	-
	Sergipe (Non-capital)	9.08	33.35	37.04	0.78	-	0.44	2.87	1.08	3.99
Southeast	Espírito Santo (Non-capital)	5.67	6.52	12.89	0.72	-	-	0.84	0.69	-
	Minas Gerais (Non-capital)	16.64	29.49	54.39	58.64	0.14	0.08	10.57	5.72	2.51
	Rio de Janeiro (Non-capital)	7.90	11.24	29.71	6.91	0.08	6.20	16.52	7.42	-
	São Paulo (Non-capital)	13.81	28.59	30.33	10.21	0.56	0.17	22.58	13.29	5.70
South	Paraná (Non-capital)	11.86	30.55	67.16	9.35	-	-	42.80	22.82	0.21
	Rio Grande do Sul (Non- capital)	19.35	11.52	24.39	7.83	6.99	0.10	11.08	10.09	-
	Santa Catarina (Non-capital)	8,64	11.70	43.46	3.97	1.13	-	28.55	5.47	0.71

Source: SIA/SUS _^(20)^_ , 2016-2022. *CAPS = Psychosocial Care Center; ^†^TRS = Therapeutic Residential Service; ^‡^ARU = Adult Reception Unit

**Table 3:** Average rates (per 10 thousand inhabitants) of CAPS* procedures offered in non-large capital municipalities, 2016 to 2022. Brazil, 2024

Region	Non-capitals	Procedures							
		Night reception	Third-shift reception	Patient follow-up in TRS^†^	Day reception	Individual care	Group care	Family care	Initial reception
Midwest	Goiás (Non-capital)	8.12	0.18	-	18.14	97.10	85.92	29.38	20.78
	Mato Grosso (Non-capital)	0.73	0.51	-	4.81	175.15	472.58	52.22	27.99
	Mato Grosso do Sul (Non-capital)	1.88	0.04	-	10.46	71.42	4.75	8.24	5.31
North	Pará (Non-capital)	15.97	0.30	-	41.27	217.30	102.62	47.12	19.31
Northeast	Alagoas (Non-capital)	0.09	0.06	-	29.43	139.54	97.42	25.52	22.19
	Bahia (Non-capital)	3.58	0.11	-	30.48	129.22	51.47	38.62	22.65
	Ceará (Non-capital)	6.99	0.10	3.57	98.70	329.65	83.86	39.74	13.56
	Maranhão (Non-capital)	1.71	0.63	0.81	105.78	35.51	93.65	17.34	16.71
	Paraíba (Non-capital)	0.17	0.06	3.50	12.92	483.69	292.65	127.90	17.50
	Pernambuco (Non-capital)	13.19	3.67	7.46	56.14	191.27	154.90	67.83	22.83
	Rio Grande do Norte (Non-capital)	14.74	-	-	81.82	185.53	231.96	26.58	14.18
Southeast	Espírito Santo (Non-capital)	0.68	0.04	-	30.85	60.50	23.54	9.82	6.88
	Minas Gerais (Non-capital)	19.61	1.86	10.39	164.55	294.96	103.72	56.44	20.94
	Rio de Janeiro (Non-capital)	4.06	0.81	3.10	44.27	136.23	83.19	27.55	9.19
	São Paulo (Non-capital)	23.56	4.03	7.09	91.52	487.80	171.91	61.98	24.01
South	Paraná (Non-capital)	9.36	4.82	3.79	34.05	260.29	240.47	78.52	21.88
	Rio Grande do Sul (Non-capital)	23.96	14.04	7.75	60.54	306.84	130.84	61.13	34.05
	Santa Catarina (Non-capital)	25.04	11.49	-	83.05	334.93	236.71	41.15	23.87
**Region**	**Non-capitals**	**Procedures**								
		**Home care**	**Body practices**	**Expressive practices**	**Care in crisis situation**	**Harm reduction actions**	Follow- up in the TRS^†^ by CAPS*	**Psychosocial rehabilitation actions**	**Promotion of contractuality**	**Follow-up of adults in an ARU^‡^**
Midwest	Goiás (Non-capital)	12.15	26.97	31.50	16.49	2.64	0.65	26.79	19.70	-
	Mato Grosso (Non-capital)	5.81	61.34	9.04	2.95	-	-	5.84	0.65	-
	Mato Grosso do Sul (Non-capital)	6.68	1.7	4.55	0.49	4.73	-	4.14	0.42	-
North	Pará (Non-capital)	5.04	45.22	22.77	4.80	0.22	-	3.12	1.77	-
Northeast	Maranhão (Non-capital)	1.17	10.84	32.30	5.62	-	0.14	59.78	5.10	-
	Alagoas (Non-capital)	2.50	1.73	2.32	4.82	-	0.43	0.19	0.85	-
	Bahia (Não capital)	2.58	5.21	7.74	6.48	23.48	0.53	10.37	3.85	-
	Ceará (Non-capital)	6.08	18.43	17.58	20.08	3.08	2.12	1.27	8.69	6.22
	Paraíba (Non-capital)	11.28	22.20	82.76	2.27	-	-	10.93	2.59	-
	Pernambuco (Non-capital)	3.67	10.14	26.38	33.07	-	0.02	8.43	7.44	0.11
	Rio Grande do Norte (Non-capital)	1.18	11.39	1.30	0.14	-	-	0.16	-	-
Southeast	Espírito Santo (Non-capital)	0.49	3.74	12.94	3.20	-	-	2.60	1.57	-
	Minas Gerais (Non-capital)	6.76	19.43	40.10	21.67	0.04	0.04	15.33	11.85	0.61
	São Paulo (Non-capital)	10.31	23.94	34.89	28.70	0.18	0.06	34.48	34.37	0.61
	Rio de Janeiro (Non-capital)	2.04	19.33	38.57	3.38	3.03	0.32	10.82	3.67	-
South	Paraná (Non-capital)	14.21	16.82	64.14	6.96	-	-	22.43	17.17	3.76
	Rio Grande do Sul (Non-capital)	7.66	21.74	40.73	10.90	4.33	0.08	16.81	7.79	2.34
	Santa Catarina (Non-capital)	13.79	14.14	49.14	10.36	3.58	-	25.11	23.68	-

Source: SIA/SUS _^(20)^_ , 2016 - 2022. *CAPS = Psychosocial Care Center; ^†^TRS = Therapeutic Residential Service; ^‡^ARU = Adult Reception Unit

In addition, the comparison between the records of the procedures offered in CAPS in the capitals and non-capitals shows that the highest averages of records are for day reception, individual care and group care (Table 4).

**Table 4:** Comparison between the average rates (per 10 thousand inhabitants) of the provision of procedures in CAPS* capitals and non-capitals, with a population above 100 thousand inhabitants, 2016 to 2022. Brazil, 2024

Procedures performed at CAPS*	Average number of records in the time series (2016 to 2022)	
	Capitals (n^†^=26)	Non-capitals (n^†^=286)
Night reception of patients in CAPS*	16.82	12.68
Third-shift reception of patients in CAPS*	3.96	4.29
Patient follow-up in TRS^†^	4.75	18.18
Day reception of patients in CAPS*	68.76	106.28
Individual patient care in CAPS*	204.48	296.77
Patient group care in CAPS*	82.62	176.49
Family care in CAPS*	39.19	56.53
Initial reception by CAPS*	19.03	23.69
Home care for CAPS patients*	4.55	9.70
Body practices in CAPS*	9.22	28.41
Expressive and communicative practices in CAPS*	21.26	36.68
Care in crisis situation	15.51	16.25
Harm reduction actions	0.39	6.39
Follow-up in the TRS^‡^ by CAPS*	1.76	0.83
Psychosocial rehabilitation actions	12.78	21.6
Promotion of contractuality	6.64	15.07
Follow-up of adults with suffering or mental disorders resulting from the use of crack, alcohol and other drugs - ARU^§^	1.15	1.74

Source: SIA/SUS^20^, 2016-2022. *CAPS = Psychosocial Care Center; ^†^n = Number of participants; ^‡^TRS = Therapeutic Residential Service; ^§^ARU = Adult Reception Unit

A significant correlation was observed between socioeconomic indicators and the provision of the following procedures: daytime reception; group care; family care; home care; body practices; expressive and communicative practices; attention in crisis situations; and promotion of contractuality (Table 5).

**Table 5:** Analysis of the correlation between the provision of procedures in Psychosocial Care Centers and socioeconomic indicators of medium and large municipalities in Brazil, 2016 to 2022

	Correlation analysis	Theil-L index	Gini index	Discharge rate	IDHM*	Illiteracy rate	***Per capita* income**	Proportion of poor
Night reception of patient in CAPS^†^	*r*_*s*_^‡^	0.204	0.209	-0.058	0.191	-0.075	0.191	-0.079
	*p-value* ^*§*^	0.003	0.002	0.401	0.005	0.274	0.005	0.246
Day reception of patients in CAPS^†^	*r*_*s*_^‡^	0.019	0.028	-0.017	0.205	-0.137	0.167	-0.141
	*p-value* ^*§*^	0.740	0.626	0.767	<0.001	0.016	0.003	0.013
Individual patient care in CAPS ^†^	*r*_*s*_^‡^	0.035	0.043	-0.167	0.165	-0.116	0.148	-0.144
	*p-value* ^*§*^	0.535	0.452	0.003	0.004	0.041	0.009	0.011
Patient group care in CAPS^†^	*r*_*s*_^‡^	-0.138	0.127	-0.136	0.200	-0.178	0.171	-0.230
	*p-value* ^*§*^	0.015	0.025	0.017	<0.001	0.002	0.003	<0.001
Family care^†^	*r*_*s*_^‡^	-0.008	0.003	-0.067	0.168	-0.154	0.161	-0.158
	*p-value* ^*§*^	0.889	0.960	0.239	0.003	0.007	0.005	0.005
Home care for patients^†^	*r*_*s*_^‡^	-0.095	-0.076	-0.267	-0.166	0.235	0.296	-0.288
	*p-value* ^*§*^	0.098	0.184	<0.001	<0.001	<0.001	<0.001	<0.001
Expressive and communicative practices	*r*_*s*_^‡^	-0.052	-0.043	-0.130	0.144	-0.155	0.172	-0.219
	*p-value* ^*§*^	0.371	0.467	0.026	0.014	0.008	0.003	<.001
Care in crisis situations	*r*_*s*_^‡^	0.090	0.104	-0.046	0.194	-0.129	0.171	-0.132
	*p-value* ^*§*^	0.123	0.074	0.433	<.001	0.027	0.003	0.023
Psychosocial rehabilitation actions	*r*_*s*_^‡^	0.023	0.038	-0.170	0.285	-0.238	0.288	-0.271
	*p-value* ^*§*^	0.700	0.520	0.004	<.001	<.001	<.001	<.001
Promotion of contractuality	*r*_*s*_^‡^	0.070	0.082	-0.126	0.210	-0.189	0.238	-0.232
	*p-value* ^*§*^	0.251	0.002	0.038	<0,001	0.002	<0.001	<0.001

Source: SIA/SUS^20^, 2016-2022; PNUD^21^, 2010; *The Jamovi Project* ^23^. *IDHM = Municipal Human Development Index; ^†^CAPS = Psychosocial Care Center; ^‡^ *rs =* Spearman’s rank correlation coefficient; ^§^p-value = Probability of significance

Regarding the correlations, it is demonstrated that the performance of the aforementioned procedures is influenced by the analyzed indicators presented with positive values, i.e., records of procedures increase with the indicator. In addition, there was a negative correlation, presented through an inversely proportional relationship.

The analysis using the Kruskal-Wallis test (non-parametric ANOVA) showed statistically significant differences between medium and large municipalities for the procedures: night care (χ²=7.26; p = 0.007), group care (χ²=4.27; p = 0.039), family care (χ² = 7.07; p = 0.008), care in crisis situations (χ²=7.20; p = 0.007) and follow-up of TRS by CAPS (χ² = 12.10; p= < 0.001).

The multiple comparisons showed statistical significance, indicating that large municipalities have lower rates when compared to medium-sized municipalities for the procedures: night reception (W = - 3.81; p = 0.007), group care (W = - 2.92, p = 0.039), family care (W = -2.92; p = 0.008) and care in crisis situations (W = -3.79; p = 0.007). Whereas the TRS follow-up procedure showed significantly higher values in large municipalities (W = 4.92; p=<0.001).

## Discussion

The findings indicate the relationship between socioeconomic factors and the procedures performed in Brazilian community-based services, considering the influence of social and economic disparities on access to and guarantee of care in services^25^. Although the correlations observed show low magnitude, they point to the growth of records in municipalities with better socioeconomic indicators, such as those with higher IDHM and *per capita* income values, which presented positive correlations.

On the other hand, negative correlations were identified, especially with the illiteracy rate indicator, evidenced by the increase in registrations to the detriment of the lower values of the indicator. In this context, other productions^26^ ^-^ ^27^ have shown that individuals with less education have low numbers of access to primary care, causing a late search for health services. Thus, the absence of health literacy, influenced by several factors, including schooling, hinders access and continuity of care^28^.

By observing the rates of provision of procedures, a national pattern is identified in the records, so that harm reduction procedures, TRS follow-up, and follow-up of people in ARU were reduced both in capitals and in medium and large non-capitals. It is noteworthy that, among these procedures, only the follow-up of people in ARU showed a statistically significant correlation with socioeconomic indicators; however, the reduced record may limit the accuracy of the analysis.

The limited registration of procedures related to services that support mental health care, such as the SRT and UAA, are related to the management of care of greater care complexity, observing the organizational characteristics. However, it is observed that the care provided to residents in the TRS occurs in a unidirectional manner, with only the displacement of users to the reference CAPS, since the follow-up of people living in the TRS has high records, while there is little record of the activities performed in the TRS. It is emphasized that the relationship between the services should be close, since the CAPS acts as a reference service for therapeutic residences^29^.

The absence of records for procedures related to the communication of the CAPS with other network devices demonstrates the limitation of care provided to the network specialized in mental health, which can result in the restriction of the transit of users through the different levels of care and in their chronicity in the service^15^. In addition, the lack of a unified referral and counter-referral system that communicates Primary Health Care, combined with the limited number of PHC teams trained to deal with mental health demands, overloads the CAPS and exposes structural and human barriers^30^ ^-^ ^31^.

The significant difference in the supply of procedures according to population size, indicating larger municipalities with lower rates, may be due to the greater capillarity of the network in larger municipalities, whose mental health offer can be carried out in other services due to the existence of a more consolidated network^32^. In addition, there is a trend towards the internalization of mental health care in medium and small cities, mainly with the participation of PHC^33^. This point is evidenced by the evolution of the CAPS coverage indicator/100 thousand inhabitants presented by the Mental Health in Data report^34^, which mentions several municipalities in the interior with CAPS coverage above 50%.

The records of the procedures have significant gaps, contributing to the weakening of the data and the lack of concrete knowledge of the offer of procedures in mental health services. In this context, the action of recording is often related only to the bureaucratic activity of the work process^35^. In addition, the professionals believe that the difficulties related to registration would be minimized if the training process were strengthened; therefore, the relevance of unified and periodic qualifications aligned with health policies^36^. It is noteworthy that the consolidation of the culture of recording, monitoring and evaluating mental health indicators qualifies the production of evidence to support decisions consistent with the needs of the population^37^.

The limitation of the study is linked to the restrictions inherent to the design of the research and the use of secondary data. The incompleteness of the data made available by the services of the municipalities in the sample in the Information Systems is highlighted, which made it impossible to carry out a more specific analysis by municipality and required the need to aggregate, by arithmetic average, the historical series. In addition, the data are generated cumulatively by municipality, and it is not possible to identify and differentiate the type of CAPS that recorded the procedure. In this sense, the absence of records makes it difficult to establish the strategic planning of the Psychosocial Care Network and to know its characteristics.

However, it is important to highlight that this research sought to contribute to the debate by discussing the provision of procedures through a socioeconomic perspective, observing the multifactoriality that affects the work process in the CAPS, without disregarding the characteristics of the territoriality of the service. It also suggests future studies based on Cluster Analysis, to analyze the groups of municipalities based on their common characteristics.

## Conclusion

A correlation was evidenced between the procedures performed and recorded in the CAPS and the socioeconomic indicators, indicating multifactoriality in the provision of care to people with MD. The close relationship between supply and income-related indicators demonstrates that the registration of the procedure follows the growth of the indicator. In this aspect, the CAPS work process should be thought of as linked to holistic care, observing the living conditions in the territory.

Therefore, there is an urgent need for strategies that minimize inconsistencies in the records, such as periodic audits to monitor the quality of information, establishment of operational protocols that guide a registration flow, and the continuous training of professionals through Permanent Health Education, so that the data are reliable to the activities carried out and guarantee tools to support public policies.

## Data Availability

All data generated or analysed during this study are included in this published article.
