# Supplementary material for: Relationship between the provision of procedures in psychosocial care centers and socioeconomic factors: an ecological study
Source: Rev Lat Am Enfermagem. 2026 Nov 13;34:e4979. doi: 10.1590/1518-8345.8045.4979 (PMC13762697; doi:10.1590/1518-8345.8045.4979)
Supplement: Translation

# Translation: Relação entre oferta de procedimentos dos centros de atenção psicossocial e fatores socioeconômicos: estudo ecológico^*^

**DOI:** 10.1590/1518-8345.8045.4980

**Authors:** Anna Helen Silva Lima, Ardigleusa Alves Coelho, Gabriela Maria Cavalcanti Costa, Ricardo Alves de Olinda, Valdecir Carneiro da Silva

**Affiliations:** 1 Universidade Estadual da Paraíba, Departamento de Enfermagem, Campina Grande, PB, Brasil.; 2 Bolsista do Conselho Nacional de Desenvolvimento Científico e Tecnológico (CNPq), Brasil.; 3 Universidade Estadual da Paraíba, Departamento de Estatística, Campina Grande, PB, Brasil.

**Keywords:** Saúde Mental, Centro de Atenção Psicossocial, Serviços Comunitários de Saúde Mental, Acesso à Saúde, Sistemas de Informação em Saúde, Gestão em Saúde.

## Abstract

**Objetivo:**

analisar a correlação entre a oferta de procedimentos realizados nos Centros de Atenção Psicossocial e os indicadores socioeconômicos nos municípios com porte populacional acima de 100 mil habitantes.

**Método:**

trata-se de estudo ecológico com abrangência de 312 municípios e 1.127 serviços. Na modelagem, extraíram-se dados da série histórica da produção ambulatorial do Sistema de Informação em Saúde e de indicadores socioeconômicos do Atlas de Desenvolvimento Humano.

**Resultados:**

apenas 12 capitais registraram ações de redução de danos; 93% dos registros são mais frequentes nas capitais. Foi observada correlação positiva entre atendimento domiciliar e renda *per capita*; e correlação negativa entre reabilitação psicossocial e taxa de analfabetismo. Destaca-se que o Índice de Desenvolvimento Humano Municipal e a renda *per capita* apresentaram correlação com a maioria dos procedimentos, enquanto a taxa de analfabetismo correlacionou-se com 64,7% deles. Evidenciaram-se diferenças estatisticamente significativas entre os municípios de médio e grande porte.

**Conclusão:**

a relação entre os procedimentos registrados dos Centros de Atenção Psicossocial e os indicadores socioeconômicos pode indicar a influência de questões multifatoriais no cuidado em saúde mental.

## Destaques

**(1)** O cuidado em Saúde Mental no Brasil sofre influência das desigualdades sociais.

**(2)** O cuidado nos Centros Comunitários de Saúde Mental concentra-se em grandes centros.

**(3)** Ausência de notificação de registros dos procedimentos nos Sistemas de Informação.

**(4)** Os procedimentos dos CAPS estão correlacionados principalmente com IDHM e renda.

**(5)** A oferta de procedimentos dos CAPS está relacionada aos fatores socioeconômicos.

## Introdução

Os transtornos mentais (TM) configuram um problema de saúde pública de ordem global e figuram entre as principais causas não fatais de morte precoce, contribuindo para a redução da expectativa de vida em cerca de 20 anos em comparação à população geral^1^. Entretanto, apenas 12% das pessoas acometidas possuem acesso aos serviços de saúde nos países de baixa renda, de acordo com o Relatório Mundial sobre Saúde Mental^2^ ^-^ ^3^. No Brasil, o financiamento destinado à saúde mental mostra-se insuficiente para atender às necessidades assistenciais, representando apenas 1,6% dos recursos totais em 2016^3^. O fortalecimento dos serviços comunitários de saúde mental está previsto no Plano de Ação Integral de Saúde Mental (2013 - 2030), que propõe o aumento de instalações em 80% dos países como estratégia para redução das taxas de reinternação psiquiátrica^2^ ^-^ ^5^.

A reforma psiquiátrica brasileira reestruturou o cuidado em saúde mental, abrangendo desde a regulamentação de direitos até a oferta de uma atenção de base comunitária^3^. Nesse contexto, Centros de Atenção Psicossocial (CAPS), regulamentados pelas Portarias do Ministério da Saúde n° 224/1992^6^ e n° 336/2002^7^, constituem serviços de referência territorial, de caráter aberto, voltados à promoção da autonomia e à (re)inserção social dos usuários^8^. Entre 1998 e 2020, o número de CAPS aumentou de 148 para 2.657 CAPS^9^, consolidando-se como componentes de Ação Estratégica da Rede de Atenção Psicossocial (RAPS) e como dispositivos substitutivos ao modelo manicomial, assegurando o trânsito dos usuários por serviços das diversas complexidades^1^ ^,^ ^10^. A partir de 2016, contudo, o cenário político nacional passou a impactar negativamente a operacionalização dos CAPS, devido à adoção de estratégias neoliberais e conservadoras que reduziram o financiamento e desestruturaram a Política Nacional de Saúde Mental (PNSM) por meio da divulgação de documentos normativos e edição de notas técnicas^4^ ^,^ ^11^. 

As atividades realizadas nos CAPS são registradas conforme a padronização de procedimentos baseada na Tabela do Sistema de Gerenciamento de Procedimentos, Medicamentos, Órteses, Próteses e Materiais Especiais do Sistema Único de Saúde (SIGTAP), registrados utilizando instrumentos como o Registro das Ações Ambulatoriais de Saúde - Psicossocial (RAAS - Psicossocial), Boletim de Produção Ambulatorial Consolidado (BPA-C) e Boletim de Produção Ambulatorial Individualizado (BPA-I), agrupados nos Sistemas de Informação em Saúde^12^. As informações fornecidas não são utilizadas para fins de financiamento, visto que o repasse financeiro dos CAPS é fixo, dependendo da sua tipologia^13^.

No entanto, ainda não há consenso acerca dos objetos de avaliação de serviços de saúde mental no Brasil, em razão da necessidade de contemplar as subjetividades que envolvem a produção de cuidado^14^. Nos CAPS, os instrumentos de avaliação utilizados frequentemente concentram-se na satisfação dos usuários e na percepção dos profissionais por meio de escalas - Avaliação da Satisfação dos Pacientes com os Serviços de Saúde Mental (SATIS-BR) e Avaliação do Impacto do Trabalho em Serviços de Saúde Mental (Impacto-BR)^15^. Sendo assim, a avaliação e acompanhamento do serviço atua como ferramenta crucial para garantir serviços efetivos e eficientes, por meio da qualificação do acesso e atendimento, além de assegurar que não reincida os cenários de negligência outrora presenciados^16^ ^-^ ^17^. 

Apesar da influência das condições socioeconômicas sobre o adoecimento e o acompanhamento do cuidado^18^, a análise da relação entre os procedimentos realizados nos CAPS e os indicadores socioeconômicos municipais permanece incipiente no cenário científico. A avaliação de dados secundários, historicamente empregados predominantemente para fins administrativos de organização de equipe, possibilita uma compreensão mais abrangente das iniquidades que permeiam a oferta do cuidado, na medida em que permite a identificação de padrões assistenciais em distintos contextos territoriais. No âmbito da saúde mental, os dados em saúde possibilitam o acompanhamento e avaliação dos serviços e das políticas públicas, considerando as especificidades socioeconômicas regionais^13^.

Portanto, considerando o quantitativo populacional estabelecido para a instalação das diferentes modalidades de CAPS, o estudo optou por avaliar os municípios de médio e grande porte, que são os principais responsáveis pela migração pendular em busca de serviços de saúde^19^. Isto posto, objetiva-se analisar a correlação entre a oferta de procedimentos realizados nos CAPS e os indicadores socioeconômicos nos municípios com porte populacional acima de 100 mil habitantes, para que, assim, possa-se produzir inferências sobre a equidade no acesso aos serviços de atendimento de base comunitária para as pessoas com transtornos mentais e comportamentais no Brasil.

## Método

### Tipo de estudo

Trata-se de um desenho de estudo ecológico, fundamentado na análise de dados secundários disponibilizados no Sistema de Informação Ambulatorial do Departamento de Informática do Sistema Único de Saúde (SIA/SUS)^20^ e no Atlas do Desenvolvimento Humano no Brasil, do Programa das Nações Unidas para o Desenvolvimento (PNUD)^21^. A condução do estudo observou as recomendações do *Strengthening the Reporting of Observational Studies in Epidemiology* (STROBE).

### Cenário da pesquisa 

A pesquisa abrangeu 312 municípios brasileiros, selecionados com base na estratificação populacional - acima de 100 mil habitantes, conforme dados do Censo 2022^22^. Incluindo as 26 capitais, o Distrito Federal e os demais municípios, distribuídos nas cinco regiões: 27 municípios da Região Norte, 63 do Nordeste, 143 do Sudeste, 55 do Sul e 24 do Centro-Oeste. A seleção dos municípios baseada na estratificação populacional decorreu da significativa lacuna dos dados. Neste contexto, foi analisada a produção ambulatorial de um percentual de 1.127 CAPS (40%), independentemente de sua tipologia, de um total de 2.836 (100%) existentes no país^22^. A distribuição geográfica dos CAPS incluídos na amostra acontece da seguinte forma: 53 localizados na Região Norte, 198 no Nordeste, 596 no Sudeste, 197 no Sul e 83 no Centro-Oeste.

### População e amostra 

A amostragem foi constituída por 23 procedimentos registrados em três instrumentos utilizados nas rotinas de atendimentos dos CAPS, sendo: RAAS - Psicossocial, BPA-C e BPA-I. Considerando 15 procedimentos registrados no RAAS - Psicossocial: acolhimento noturno de paciente em CAPS; acolhimento em terceiro turno de paciente em CAPS; acolhimento diurno de paciente em CAPS; atendimento individual de paciente em CAPS; atendimento em grupo de paciente em CAPS; atendimento familiar em CAPS; atendimento domiciliar para paciente de CAPS; práticas corporais em CAPS; práticas expressivas e comunicativas em CAPS; atenção às situações de crise; ações de reabilitação psicossocial; promoção de contratualidade; acompanhamento de pessoas adultas com sofrimento ou transtornos mentais decorrentes do uso de *crack*, álcool e outras drogas - Unidade de Acolhimento Adulto e Acompanhamento da população infantojuvenil com sofrimento ou transtornos mentais decorrentes do uso de *crack*, álcool e outras drogas - Unidade de Acolhimento Infantojuvenil. Enquanto são registrados no BPA-C um total de sete procedimentos, dos quais: ações de articulação de redes intra e intersetoriais; fortalecimento de protagonismo de usuário de CAPS e seus familiares; matriciamento de equipes da atenção básica; ações de redução de danos; acompanhamento de serviço residencial terapêutico por CAPS; apoio ao serviço residencial de caráter transitório por CAPS; matriciamento de equipes de pontos de atenção da urgência e emergência e dos serviços hospitalares de referência para atenção a pessoas com sofrimento ou transtorno mental e com necessidades de saúde decorrentes do uso de álcool, *crack* e outras drogas. Por fim, o acolhimento inicial por CAPS, registrado no BPA-I.

Considerou-se como critério de inclusão a existência de registros presentes em pelo menos 10% dos 312 municípios analisados, foram eliminados cinco procedimentos: Ações de articulação de redes intra e intersetoriais; fortalecimento do protagonismo de usuários de CAPS e seus familiares; Matriciamento de equipes de Atenção Básica; Acompanhamento da população infantojuvenil com sofrimento ou transtornos mentais decorrentes do uso de *crack*, álcool e outras drogas - Unidade de Acolhimento Infantojuvenil (UAI) e matriciamento de equipes de pontos de atenção da urgência e emergência e dos serviços hospitalares de referência para atenção a pessoas com sofrimento ou transtorno mental e com necessidades de saúde decorrentes do uso de álcool, crack e outras drogas. A amostra final foi constituída por 18 procedimentos. Para os municípios, o critério de inclusão observado foi a existência de registros de procedimentos em pelo menos um ano da série histórica e população superior a 100 mil. 

### Procedimentos e instrumentos para coleta de dados 

A coleta de dados foi realizada durante o período de novembro de 2023 e agosto de 2024, por meio de duas etapas, sendo: (1) por meio do Sistema de Informação Ambulatorial do Departamento de Informática do Sistema Único de Saúde^20^ (https://datasus.saude.gov.br); e (2) por meio do Atlas de Desenvolvimento Humano no Brasil^21^ ^)^ (https://www.atlasbrasil.org.br). 

Na etapa 1, inicialmente, foram coletados dados secundários obtidos pelo SIA/SUS sobre os registros de procedimentos (variáveis dependentes) em CAPS habilitados pelo Ministério da Saúde que apresentavam a produção ambulatorial por meio dos instrumentos RAAS, BPA-C e BPA-I. 

Para a etapa 2, as variáveis independentes foram os indicadores socioeconômicos disponibilizados no Atlas de Desenvolvimento Humano no Brasil: Índice de Desenvolvimento Humano Municipal (IDHM), índice de Gini, índice de Theil L, taxa de desocupação (população com 18 anos ou mais), taxa de analfabetismo (população com 15 anos ou mais), renda *per capita* (salário mínimo) e proporção de pobres.

### Procedimentos de análise dos dados

Inicialmente, foi realizada a análise descritiva dos dados com cálculos e comparações de média de oferta nos procedimentos nos CAPS, considerando grupos de municípios com população entre 100.000 e 199.999 (médio porte) e os maiores que 200.000 habitantes (grande porte). Para as variáveis dependentes, foi calculada a média aritmética simples da série histórica (2016 a 2022) por município. Os dados referentes à oferta de procedimentos foram padronizados para comparação entre os grupos de municípios com base no quantitativo populacional, mediante cálculo da média de procedimentos da série histórica pelo número de habitantes e multiplicando o resultado por 10.000 habitantes.

Após isso, utilizou-se o *software* gratuito Jamovi, versão 2.3^23^, para aplicação dos testes estatísticos. Primeiramente, realizou-se o teste de Shapiro-Wilk, indicando que os dados são não paramétricos. Realizou-se o teste estatístico de correlação de Spearman para avaliar a oferta de procedimentos realizados no CAPS e os indicadores socioeconômicos. Adicionalmente, foi realizada a ANOVA não paramétrica de Kruskal-Wallis para comparação entre a oferta de procedimentos entre os grupos de municípios segundo o porte. Adotou-se significância estatística em 5%, ou seja, valor de p<0,05.

### Aspectos éticos

De acordo com a Resolução do Conselho Nacional de Saúde n.º 510/2012^24^, dispensa-se a submissão de pesquisas quando utilizam dados de domínio público e acesso aberto.

## Resultados

A distribuição dos procedimentos ofertados por municípios ocorreu da seguinte forma: atendimento individual (100%), atendimento familiar (99,6%), atendimento em grupo (99,3%), acolhimento diurno (99%), acolhimento inicial (98,7%), atendimento domiciliar (97,7%), atenção em situação de crises (94,5%), práticas expressivas e comunicativas (94,2%), práticas corporais (93,2%), ações de reabilitação psicossocial (92,9%), promoção da contratualidade (86,8%), acolhimento noturno (68,9%), acolhimento em terceiro turno (53,8%), acompanhamento de paciente em Serviço Residencial Terapêutico (28,2%), acompanhamento de Serviço Residencial Terapêutico (20,1%), ações de redução de danos (15%) e acompanhamento de pessoas adultas em Unidade de Acolhimento Adulto (10%).

Na Tabela 1, é possível identificar a taxa (por 10 mil habitantes) de oferta dos procedimentos nos CAPS segundo a produção ambulatorial nas capitais brasileiras. Destacam-se registros de procedimentos com taxas elevadas para capitais das seguintes regiões: Centro-Oeste (Brasília e Campo Grande); Norte (Palmas e Rio Branco); Nordeste (João Pessoa e Recife); Sudeste (São Paulo e Vitória); e Sul (Curitiba e Porto Alegre).

**Tabela 1:** Taxas (por 10 mil hab.) de oferta de procedimentos nos CAPS* em capitais brasileiras, 2016 a 2022. Brasil, 2024

Região	Capitais	Procedimentos							
		Acolhimento noturno	Acolhimento em terceiro turno	Acomp. de paciente em SRT^†^	Acolhimento diurno	Atend. individual	Atend. em grupo	Atend. familiar	Acolhimento inicial
Centro-Oeste	Brasília	1,02	0,43	-	18,21	102,2	33,72	16,13	16,62
	Campo Grande	68,24	0,03	1,44	76,82	508,44	142,78	74,34	27,98
	Cuiabá	-	-	-	4,79	95,78	55,46	39,55	20,62
	Goiânia	0,12	0,10	1,58	2,54	63,21	29,33	13,53	7,14
Norte	Boa Vista	6,95	16,12	-	-	61,70	39,31	5,65	25,52
	Belém	1,68	-	-	5,00	186,25	17,56	53,64	19,32
	Macapá	-	-	-	6,21	47,80	28,82	10,34	-
	Manaus	1,12	-	-	3,33	123,94	11,69	35,70	12,85
	Palmas	14,25	1,12	-	56,19	555,70	182,20	70,57	62,88
	Rio Branco	12,76	-	-	20,47	230,34	10,72	3,30	9,99
Nordeste	Aracaju	18,79	0,17	15,80	74,63	153,33	66,10	63,67	9,55
	Fortaleza	0,11	0,55	0,22	16,92	78,90	11,51	2,13	24,40
	João Pessoa	11,92	11,98	-	302,87	101,04	65,84	7,17	2,92
	Maceió	7,81	2,22	0,01	97,06	169,31	141,31	39,29	14,37
	Natal	10,88	-	0,96	3,53	138,00	119,50	10,50	10,05
	Recife	24,61	8,18	20,25	75,77	285,35	199,50	84,83	30,16
	Salvador	0,41	0,01	0,47	74,21	113,84	41,04	21,98	6,08
	São Luís	0,88	0,01	-	109,33	115,68	57,30	30,47	2,69
	Teresina	26,15	-	2,07	63,90	275,90	145,82	116,50	6,86
Sudeste	Belo Horizonte	34,17	15,23	11,90	256,55	193,48	11,95	1,09	4,11
	Rio de Janeiro	7,37	6,19	4,01	45,55	120,99	24,61	28,76	8,83
	São Paulo	26,41	6,10	1,56	116,68	327,15	147,52	70,46	33,95
	Vitória	38,03	0,13	-	226,32	296,81	86,11	45,49	36,23
Sul	Curitiba	87,79	2,52	1,43	81,14	661,59	311,37	131,73	50,66
	Florianópolis	0,02	0,03	-	10,67	172,57	196,33	16,70	32,33
	Porto Alegre	17,33	8,04	-	32,33	224,33	45,57	33,83	18,54
**Região**	**Capitais**	**Procedimentos**								
		**Atendim. domiciliar**	**Práticas corporais**	**Práticas expressivas**	**Atenção às situações de crise**	**Ações de red. de danos**	Acomp. de SRT^†^ por CAPS*	**Ações de reabili. psicossocial**	**Promoção da contratualidade**	**Acomp. de pessoas adultas em UAA^‡^**
Centro-Oeste	Brasília	0,76	2,89	398	6,64	0,01	0,04	9,15	1,82	-
	Campo Grande	1,25	13,16	28,53	80,25	-	-	3,82	3,50	1,11
	Cuiabá	5,08	0,41	20,15	0,86	0,12	-	1,35	1,25	-
	Goiânia	0,53	5,10	3,80	0,92	0,02	0,03	1,98	1,79	-
Norte	Belém	1,33	10,05	9,20	3,02	-	0,21	9,20	11,23	-
	Boa Vista	1,03	5,84	1,17	2,44	0,10	0,46	-	0,05	0,86
	Macapá	0,05	0,05	2,22	3,57	-	-	0,07	0,04	-
	Manaus	0,88	6,69	6,12	2,01	-	0,14	6,12	7,47	-
	Palmas	18,75	13,06	5,15	2,63	0,36	0,45	13,89	0,51	-
	Porto Velho	4,64	32,51	8,41	7,13	0,10	-	3,43	0,34	-
	Rio Branco	0,56	0,03	0,82	-	0,59	-	6,13	7,68	4,62
Nordeste	Aracaju	9,72	10,71	36,19	41,22	-	-	38,11	7,80	1,36
	Fortaleza	0,66	0,20	0,14	0,28	-	0,04	0,59	0,04	0,16
	João Pessoa	1,40	1,92	25,89	4,70	-	26,70	3,97	0,06	-
	Maceió	4,55	13,03	15,94	4,23	-	0,01	4,86	1,00	-
	Natal	0,14	0,95	2,76	2,07	-	0,01	0,79	0,24	-
	Recife	11,13	6,13	17,35	22,20	0,12	0,01	8,74	26,45	1,85
	Salvador	2,09	10,81	30,78	1,89	-	-	6,42	4,32	-
	São Luís	11,61	20,13	25,54	37,17	-	-	19,02	0,30	-
	Teresina	7,78	10,45	35,61	58,18	-	-	7,68	-	-
Sudeste	Belo Horizonte	12,18	4,46	3,61	5,75	0,01	-	0,88	5,02	-
	Rio de Janeiro	2,75	6,98	22,00	12,21	1,08	0,03	11,63	6,81	-
	São Paulo	14,65	29,84	85,10	14,00	-	-	49,28	24,52	0,38
	Vitória	3,12	3,38	112,34	8,49	-	-	19,45	10,83	-
Sul	Curitiba	4,75	28,03	44,43	68,98	-	0,02	80,13	36,37	-
	Florianópolis	0,06	4,47	16,26	5,53	-	-	3,23	6,27	-
	Porto Alegre	0,91	9,70	10,41	6,58	2,56	0,01	22,50	6,89	0,01

Fonte: SIA/SUS _^(20)^_ , 2016-2022. *CAPS = Centro de Atenção Psicossocial; ^†^SRT = Serviço Residência Terapêutico; ^‡^UAA = Unidade de Acolhimento Adulto

Nesse contexto, conforme apresentado na Tabela 2, elaborada com base na oferta de procedimentos em não capitais de médio porte, destaca-se que determinadas Unidades Federativas não possuem ocorrências para municípios não capitais na amostra, uma vez que os municípios do interior não apresentam o porte populacional estabelecido na pesquisa.A Tabela 3 apresenta o cenário das não-capitais com municípios de grande porte. Observam-se ocorrências com taxas elevadas para as seguintes regiões e não capitais dos estados: Centro-Oeste (Mato Grosso e Mato Grosso do Sul); Norte (Rondônia e Pará); Nordeste (Maranhão e Paraíba); Sudeste (Minas Gerais e São Paulo); e Sul (Paraná e Santa Catarina).

Cabe mencionar que procedimentos como acolhimento em terceiro turno e acolhimento noturno concentram-se nesse grupo, uma vez que estão restritos a serviços que funcionam de maneira ininterrupta, 24 horas por dia. Portanto, a inexistência de registros desses procedimentos nos municípios não capitais de médio porte é um achado esperado no conjunto dos dados analisados.

**Tabela 2:** Média das taxas (por 10 mil hab.) de oferta de procedimentos nos CAPS* em municípios não capitais de médio porte, 2016 a 2022. Brasil, 2024

Região	Não capitais	Procedimentos							
		Acolhimento noturno	Acolhimento em terceiro turno	Acomp. de paciente em SRT^†^	Acolhimento diurno	Atend. individual	Atend. em grupo	Atend. familiar	Acolhimento inicial
Centro-Oeste	Goiás (Não capital)	0,16	0,07	1,65	15,21	129,03	67,45	6,01	18,32
	Mato Grosso (Não capital)	0,09	-	-	61,83	194,70	26,89	52,07	26,00
	Mato Grosso do Sul (Não capital)	-	-	-	50,62	151,46	0,30	6,88	19,81
Norte	Amapá (Não capital)	-	-	-	1,17	17,79	5,70	0,75	1,63
	Amazonas (Não capital)	3,23	-	-	3,94	162,54	67,71	20,14	13,63
	Pará (Não capital)	6,27	1,24	-	47,99	227,16	112,74	65,39	18,16
	Rondônia (Não capital)	0,38	-	-	58,78	138,38	2,32	0,14	0,37
Nordeste	Bahia (Não capital)	0,45	0,50	-	154,89	267,26	120,32	83,49	29,63
	Ceará (Não capital)	1,22	0,13	-	49,17	241,49	18,22	15,25	20,73
	Maranhão (Não capital)	27,80	26,40	-	816,66	843,12	209,06	84,45	114,72
	Paraíba (Não capital)	1,78	1,73	1,04	72,59	541,96	5678,58	718,45	22,35
	Pernambuco (Não capital)	12,74	18,03	12,85	18,12	170,13	186,90	110,74	19,91
	Piauí (Não capital)	-	0,98	-	0,22	28,47	4,31	0,39	0,10
	Rio Grande do Norte (Não capital)	1,58	-	-	44,49	116,76	31,17	51,25	0,11
	Sergipe (Não capital)	0,45	8,12	233,02	24,23	230,68	268,88	70,25	11,09
Sudeste	Espírito Santo (Não capital)	0,42	0,15	-	123,43	169,93	42,84	14,28	9,02
	Minas Gerais (Não capital)	26,75	7,60	73,71	169,92	424,85	127,07	63,48	35,34
	Rio de Janeiro (Não capital)	0,82	0,24	6,13	236,19	160,75	114,21	44,56	26,12
	São Paulo (Não capital)	8,11	0,42	8,88	100,14	285,34	118,81	45,71	15,60
Sul	Paraná (Não capital)	0,55	1,18	-	102,73	257,47	258,92	56,41	20,64
	Rio Grande do Sul (Não capital)	6,62	0,47	2,55	32,79	338,96	105,49	56,34	28,92
	Santa Catarina (Não capital)	0,81	27,28	-	52,52	309,46	178,26	49,80	35,74
**Região**	**Não capitais**	**Procedimentos**								
		**Atend. domiciliar**	**Práticas corporais**	**Práticas expressivas**	**Atenção às situações de crise**	**Ações de redução de danos**	Acomp. de SRT^†^ por CAPS*	**Ações de reabilitação psicossocial**	**Promoção da contratualidade**	**Acomp. de pessoas adultas em UAA^‡^**
Centro-Oeste	Goiás (Não capital)	7,07	10,56	21,94	1,85	2,53	0,20	1,30	1,42	-
	Mato Grosso (Não capital)	7,65	14,15	37,48	5,55	2,31	0,21	30,98	13,83	-
	Mato Grosso do Sul (Não capital)	1,85	-	39,09	4,82	-	-	0,90	1,32	-
Norte	Amapá (Não capital)	-	4,01	4,74	3,15	-	-	9,62	2,03	-
	Amazonas (Não capital)	2,22	0,95	7,15	5,91	1,20	-	15,21	0,51	-
	Pará (Não capital)	3,13	42,92	47,54	7,97	-	-	24,67	3,20	-
	Rondônia (Não capital)	10,44	-	-	-	-	-	17,48	2,70	-
Nordeste	Maranhão (Não capital)	1,93	30,87	22,60	47,81	-	0,60	84,58	0,09	-
	Bahia (Não capital)	20,56	55,67	80,21	2,28	0,27	-	45,86	40,45	-
	Ceará (Não capital)	6,14	1,53	0,11	2,06	-	0,08	0,28	-	-
	Paraíba (Não capital)	7,77	706,94	25,23	3,13	0,10	-	2,13	0,99	-
	Pernambuco (Não capital	6,36	8,66	33,95	45,55	-	0,47	7,40	42,62	0,63
	Piauí (Não capital)	0,09	0,06	-	-	-	-	0,31	0,06	-
	Rio Grande do Norte (Não capital)	1,42	1,65	2,66	0,08	-	-	0,55	0,17	-
	Sergipe (Não capital)	9,08	33,35	37,04	0,78	-	0,44	2,87	1,08	3,99
Sudeste	Espírito Santo (Não capital)	5,67	6,52	12,89	0,72	-	-	0,84	0,69	-
	Minas Gerais (Não capital)	16,64	29,49	54,39	58,64	0,14	0,08	10,57	5,72	2,51
	Rio de Janeiro (Não capital)	7,90	11,24	29,71	6,91	0,08	6,20	16,52	7,42	-
	São Paulo (Não capital)	13,81	28,59	30,33	10,21	0,56	0,17	22,58	13,29	5,70
Sul	Paraná (Não capital)	11,86	30,55	67,16	9,35	-	-	42,80	22,82	0,21
	Rio Grande do Sul (Não capital)	19,35	11,52	24,39	7,83	6,99	0,10	11,08	10,09	-
	Santa Catarina (Não capital)	8,64	11,70	43,46	3,97	1,13	-	28,55	5,47	0,71

Fonte: SIA/SUS _^(20)^_ , 2016-2022. *CAPS = Centro de Atenção Psicossocial; ^†^SRT = Serviço Residencial Terapêutico; ^‡^UAA = Unidade de Acolhimento Adulto

**Tabela 3:** Média das taxas (por 10 mil hab.) de ofertas de procedimentos nos CAPS* em municípios não capitais de grande porte, 2016 a 2022. Brasil, 2024

Região	Não capitais	Procedimentos							
		Acolhimento noturno	Acolhimento em terceiro turno	Acomp. de paciente em SRT^†^	Acolhimento diurno	Atend. individual	Atend. em grupo	Atend. familiar	Acolhimento inicial
Centro-Oeste	Goiás (Não capital)	8,12	0,18	-	18,14	97,10	85,92	29,38	20,78
	Mato Grosso (Não capital)	0,73	0,51	-	4,81	175,15	472,58	52,22	27,99
	Mato Grosso do Sul (Não capital)	1,88	0,04	-	10,46	71,42	4,75	8,24	5,31
Norte	Pará (Não capital)	15,97	0,30	-	41,27	217,30	102,62	47,12	19,31
Nordeste	Alagoas (Não capital)	0,09	0,06	-	29,43	139,54	97,42	25,52	22,19
	Bahia (Não capital)	3,58	0,11	-	30,48	129,22	51,47	38,62	22,65
	Ceará (Não capital)	6,99	0,10	3,57	98,70	329,65	83,86	39,74	13,56
	Maranhão (Não capital)	1,71	0,63	0,81	105,78	35,51	93,65	17,34	16,71
	Paraíba (Não capital)	0,17	0,06	3,50	12,92	483,69	292,65	127,90	17,50
	Pernambuco (Não capital)	13,19	3,67	7,46	56,14	191,27	154,90	67,83	22,83
	Rio Grande do Norte (Não capital)	14,74	-	-	81,82	185,53	231,96	26,58	14,18
Sudeste	Espírito Santo (Não capital)	0,68	0,04	-	30,85	60,50	23,54	9,82	6,88
	Minas Gerais (Não capital)	19,61	1,86	10,39	164,55	294,96	103,72	56,44	20,94
	Rio de Janeiro (Não capital)	4,06	0,81	3,10	44,27	136,23	83,19	27,55	9,19
	São Paulo (Não capital)	23,56	4,03	7,09	91,52	487,80	171,91	61,98	24,01
Sul	Paraná (Não capital)	9,36	4,82	3,79	34,05	260,29	240,47	78,52	21,88
	Rio Grande do Sul (Não capital)	23,96	14,04	7,75	60,54	306,84	130,84	61,13	34,05
	Santa Catarina (Não capital)	25,04	11,49	-	83,05	334,93	236,71	41,15	23,87
**Região**	**Não capitais**	**Procedimentos**								
		**Atend. domici-liar**	**Práticas corporais**	**Práticas expressivas**	**Atenção às situações de crise**	**Ações de redução de danos**	Acomp.de SRT^†^ por CAPS*	**Ações de reabilitação psicossocial**	**Promoção da contratualidade**	**Acomp. de pessoas adultas em UAA^‡^**
Centro-Oeste	Goiás (Não capital)	12,15	26,97	31,50	16,49	2,64	0,65	26,79	19,70	-
	Mato Grosso (Não capital)	5,81	61,34	9,04	2,95	-	-	5,84	0,65	-
	Mato Grosso do Sul (Não capital)	6,68	1,17	4,55	0,49	4,73	-	4,14	0,42	-
Norte	Pará (Não capital)	5,04	45,22	22,77	4,80	0,22	-	3,12	1,77	-
Nordeste	Maranhão (Não capital)	1,17	10,84	32,30	5,62	-	0,14	59,78	5,10	-
	Alagoas (Não capital)	2,50	1,73	2,32	4,82	-	0,43	0,19	0,85	-
	Bahia (Não capital)	2,58	5,21	7,74	6,48	23,48	0,53	10,37	3,85	-
	Ceará (Não capital)	6,08	18,43	17,58	20,08	3,08	2,12	1,27	8,69	6,22
	Paraíba (Não capital)	11,28	22,20	82,76	2,27	-	-	10,93	2,59	-
	Pernambuco (Não capital)	3,67	10,14	26,38	33,07	-	0,02	8,43	7,44	0,11
	Rio Grande do Norte (Não capital)	1,18	11,39	1,30	0,14	-	-	0,16	-	-
Sudeste	Espírito Santo (Não capital)	0,49	3,74	12,94	3,20	-	-	2,60	1,57	-
	Minas Gerais (Não capital)	6,76	19,43	40,10	21,67	0,04	0,04	15,33	11,85	0,61
	São Paulo (Não capital)	10,31	23,94	34,89	28,70	0,18	0,06	34,48	34,37	0,61
	Rio de Janeiro (Não capital)	2,04	19,33	38,57	3,38	3,03	0,32	10,82	3,67	-
Sul	Paraná (Não capital)	14,21	16,82	64,14	6,96	-	-	22,43	17,17	3,76
	Rio Grande do Sul (Não capital)	7,66	21,74	40,73	10,90	4,33	0,08	16,81	7,79	2,34
	Santa Catarina (Não capital)	13,79	14,14	49,14	10,36	3,58	-	25,11	23,68	-

Fonte: SIA/SUS _^(20)^_ , 2016 - 2022. *CAPS= Centro de Atenção Psicossocial; ^†^SRT= Serviço Residência Terapêutico; ^‡^UAA= Unidade de Acolhimento Adulto

Ademais, a comparação entre os registros dos procedimentos ofertados em CAPS nas capitais e não capitais mostra que as médias de registros mais elevadas são para acolhimento diurno, atendimento individual e atendimento em grupo (Tabela 4).

**Tabela 4:** Comparação entre média das taxas (por 10 mil habitantes) da oferta de procedimentos nos CAPS* capitais e não capitais, com população acima de 100 mil habitantes, 2016 a 2022. Brasil, 2024

Procedimentos realizados nos CAPS*	Média de registros da série histórica (2016 a 2022)	
	Capitais (n^†^=26)	Não capitais (n^†^=286)
Acolhimento noturno de paciente em CAPS*	16,82	12,68
Acolhimento em terceiro turno de paciente em CAPS*	3,96	4,29
Acompanhamento de paciente em SRT^†^	4,75	18,18
Acolhimento diurno de paciente em CAPS*	68,76	106,28
Atendimento individual de paciente em CAPS*	204,48	296,77
Atendimento em grupo de paciente em CAPS*	82,62	176,49
Atendimento familiar em CAPS*	39,19	56,53
Acolhimento inicial por CAPS*	19,03	23,69
Atendimento domiciliar para pacientes de CAPS*	4,55	9,70
Práticas corporais em CAPS*	9,22	28,41
Práticas expressivas e comunicativas em CAPS*	21,26	36,68
Atenção às situações de crise	15,51	16,25
Ações de redução de danos	0,39	6,39
Acompanhamento de SRT^‡^ por CAPS*	1,76	0,83
Ações de reabilitação psicossocial	12,78	21,6
Promoção de contratualidade	6,64	15,07
Acompanhamento de pessoas adultas com sofrimento ou transtornos mentais decorrentes do uso de *crack*, álcool e outras drogas - UAA^§^	1,15	1,74

Fonte: SIA/SUS^20^, 2016-2022. *CAPS = Centro de Atenção Psicossocial; ^†^n = Número de participantes; ^‡^SRT = Serviço Residencial Terapêutico; ^§^UAA = Unidade de Acolhimento Adulto

Observou-se correlação significativa entre indicadores socioeconômicos com a oferta dos procedimentos de: acolhimento diurno; atendimento em grupo; atendimento familiar; atendimento domiciliar; práticas corporais; práticas expressivas e comunicativas; atenção em situação de crise; e promoção da contratualidade (Tabela 5).

Acerca das correlações, demonstra-se que a realização dos procedimentos supracitados é influenciada pelos indicadores analisados apresentados com valores positivos, ou seja, registros de procedimentos aumentam com o indicador. E, ainda, ocorreu correlação negativa, apresentada por meio de uma relação inversamente proporcional.

**Tabela 5:** Análise de correlação entre oferta de procedimentos dos Centros de Atenção Psicossocial e indicadores socioeconômicos de municípios de médio e grande porte no Brasil, 2016 a 2022. Brasil, 2024

	Análise da correlação	Índice de Theil-L	Índice de Gini	Taxa de desocupação	IDHM*	Taxa de analfabetismo	**Renda *per capita***	Proporção de pobres
Acolhimento noturno de paciente em CAPS^†^	*r*_*s*_^‡^	0,204	0,209	-0,058	0,191	-0,075	0,191	-0,079
	*Valor p* ^*§*^	0,003	0,002	0,401	0,005	0,274	0,005	0,246
Acolhimento diurno de paciente em CAPS^†^	*r*_*s*_^‡^	0,019	0,028	-0,017	0,205	-0,137	0,167	-0,141
	*Valor p* ^*§*^	0,740	0,626	0,767	<0,001	0,016	0,003	0,013
Atendimento individual de paciente em CAPS ^†^	*r*_*s*_^‡^	0,035	0,043	-0,167	0,165	-0,116	0,148	-0,144
	*Valor p* ^*§*^	0,535	0.452	0,003	0,004	0,041	0,009	0,011
Atendimento em grupo de paciente em CAPS^†^	*r*_*s*_^‡^	-0,138	0,127	-0,136	0,200	-0,178	0,171	-0,230
	*Valor p* ^*§*^	0,015	0,025	0,017	<0,001	0,002	0,003	<0,001
Atendimento familiar^†^	*r*_*s*_^‡^	-0,008	0,003	-0,067	0,168	-0,154	0,161	-0,158
	*Valor p* ^*§*^	0,889	0,960	0,239	0,003	0,007	0,005	0,005
Atendimento domiciliar para pacientes^†^	*r*_*s*_^‡^	-0,095	-0,076	-0,267	-0,166	0,235	0,296	-0,288
	*Valor p* ^*§*^	0,098	0,184	<0,001	<0,001	<0,001	<0,001	<0,001
Práticas expressivas e comunicativas	*r*_*s*_^‡^	-0,052	-0,043	-0,130	0,144	-0,155	0,172	-0,219
	*Valor p* ^*§*^	0,371	0,467	0,026	0,014	0,008	0,003	<,001
Atenção às situações de crise	*r*_*s*_^‡^	0,090	0,104	-0,046	0,194	-0,129	0,171	-0,132
	*Valor p* ^*§*^	0,123	0,074	0.433	<,001	0,027	0,003	0,023
Ações de reabilitação psicossocial	*r*_*s*_^‡^	0,023	0,038	-0,170	0,285	-0,238	0,288	-0,271
	*Valor p* ^*§*^	0,700	0,520	0,004	<,001	<,001	<,001	<,001
Promoção de contratualidade	*r*_*s*_^‡^	0,070	0,082	-0,126	0,210	-0,189	0,238	-0,232
	*Valor p* ^*§*^	0,251	0,002	0,038	<0,001	0,002	<0,001	<0,001

Fonte: SIA/SUS^20^, 2016-2022; PNUD^21^, 2010; *The Jamovi Project* ^23^. *IDHM = Índice de Desenvolvimento Humano Municipal; ^†^CAPS = Centro de Atenção Psicossocial; ^‡^ *rs =* Coeficiente de correlação de postos de Spearman; ^§^Valor p = Probabilidade de significância

A análise por meio do teste de Kruskal-Wallis (ANOVA não paramétrica) evidenciou diferenças estatisticamente significativas entre os municípios de médio e grande porte para os procedimentos: acolhimento noturno (χ²=7,26; p = 0,007), atendimento em grupo (χ²=4,27; p = 0,039), atendimento familiar (χ² = 7,07; p = 0,008), atenção em situação de crise (χ²=7,20; p = 0,007) e acompanhamento de SRT por CAPS (χ² = 12,10; p= < 0,001).

As comparações múltiplas evidenciaram significância estatística, indicando que os municípios de grande porte apresentam taxas menores quando comparados aos de médio porte para os procedimentos: acolhimento noturno (W = - 3,81; p = 0,007), atendimento em grupo (W = - 2,92, p = 0,039), atendimento familiar (W = -2,92; p = 0,008) e atenção em situação de crise (W = -3,79; p = 0,007). Enquanto o procedimento acompanhamento de SRT apresentou valores significativamente maiores nos municípios de grande porte (W = 4,92; p=<0,001).

## Discussão

 Os achados indicam a relação entre os fatores socioeconômicos e os procedimentos realizados nos serviços brasileiros de base comunitária, considerando a influência das disparidades sociais e econômicas no acesso e garantia do cuidado nos serviços^25^. Embora as correlações observadas demonstrem baixa magnitude, apontam o crescimento de registros em municípios com melhores indicadores socioeconômicos, como aqueles com valores mais elevados de IDHM e renda *per capita*, que apresentaram correlações positivas.

Em contrapartida, foram identificadas correlações negativas, sobretudo com o indicador taxa de analfabetismo, evidenciado pelo crescimento de registros em detrimento aos menores valores do indicador. Nesse contexto, outras produções^26^ ^-^ ^27^ evidenciaram que indivíduos com menor escolaridade apresentam baixos números de acesso aos cuidados primários, ocasionando uma busca tardia pelos serviços de saúde. Desse modo, a ausência da literacia em saúde, influenciada por diversos fatores, entre eles a escolaridade, dificulta o acesso e a continuidade do cuidado^28^.

Ao observar as taxas de oferta de procedimentos, identifica-se um padrão nacional nos registros, de modo que os procedimentos de redução de danos, acompanhamento de SRT e acompanhamento de pessoas em UAA foram reduzidos tanto em capitais quanto em não capitais de médio e grande porte. Destaca-se que, entre esses procedimentos, apenas o acompanhamento de pessoas em UAA apresentou correlação estatisticamente significativa com os indicadores socioeconômicos; contudo, o reduzido registro pode limitar a precisão da análise. 

O registro limitado de procedimentos relacionados a serviços apoiadores do cuidado em saúde mental, como o SRT e UAA, está relacionado ao manejo de cuidado de maior complexidade assistencial, observando as características organizacionais. Contudo, observa-se que o cuidado aos residentes nas SRT acontece de maneira unidirecional, apenas com o deslocamento dos usuários até os CAPS de referência, uma vez que o acompanhamento de pessoas residentes dos SRT apresenta elevados registros, enquanto há reduzido registro das atividades realizadas nas SRT. Ressalta-se que a relação entre os serviços deve ser estreita, uma vez que o CAPS atua como serviço de referência para as residências terapêuticas^29^.

A ausência de registros para procedimentos relacionados à comunicação do CAPS com outros dispositivos de rede demonstra a limitação de atendimentos à rede especializada em saúde mental, o que pode resultar na restrição do trânsito dos usuários pelos diferentes níveis de atenção e na sua cronificação no serviço^15^. Ademais, a inexistência de um sistema unificado de referência e contrarreferência que comunique a Atenção Primária à Saúde, aliada ao número limitado de equipes de APS capacitadas para lidar com demandas de saúde mental, sobrecarrega o CAPS e expõe barreiras estruturais e humanas^30^ ^-^ ^31^.

A diferença significativa na oferta de procedimentos segundo o porte populacional, indicando municípios de maior porte apresentando menores taxas, pode se dar em função da maior capilaridade da rede nos municípios de maior porte, cuja oferta de saúde mental pode ser realizada em outros serviços devido à existência de rede mais consolidada^32^. Adiciona-se que existe, no Brasil, uma tendência à interiorização da atenção em saúde mental em cidades de médio e pequeno porte, acontecendo principalmente com a participação da APS^33^. Este ponto é evidenciado pela evolução do indicador de cobertura de CAPS/100 mil habitantes apresentado pelo relatório Saúde Mental em Dados^34^, que menciona diversos municípios interioranos com cobertura de CAPS acima de 50%.

Os registros dos procedimentos apresentam lacunas significativas, contribuindo para a fragilização dos dados e desconhecimento concreto da oferta de procedimentos nos serviços de saúde mental. Nesse contexto, a ação de registrar é frequentemente relacionada apenas à atividade burocrática do processo de trabalho^35^. Somado a isso, os profissionais acreditam que as dificuldades relacionadas ao registro seriam minimizadas se o processo de capacitação fosse fortalecido; por isso, a relevância de qualificações unificadas e periódicas alinhadas às políticas de saúde^36^. Destaca-se que a consolidação da cultura de registro, monitoramento e avaliação dos indicadores de saúde mental qualifica a produção de evidências para subsidiar decisões condizentes às necessidades da população^37^.

A limitação do estudo está vinculada às restrições inerentes ao delineamento da pesquisa e ao uso de dados secundários. Destaca-se a incompletude dos dados disponibilizados pelos serviços dos municípios da amostra nos Sistemas de Informação, o que impossibilitou uma análise mais específica por município e exigiu a necessidade de agregar, por média aritmética, a série histórica. Além disso, os dados são gerados de forma cumulativa por município, não sendo possível identificar e diferenciar a tipologia do CAPS que realizou o registro do procedimento. Nesse sentido, a ausência de registros dificulta o estabelecimento do planejamento estratégico da Rede de Atenção Psicossocial e o conhecimento de suas características. 

Contudo, é importante destacar que essa pesquisa buscou colaborar para o debate discutindo a oferta de procedimentos por meio do olhar socioeconômico, observando a multifatoriedade que atinge o processo de trabalho nos CAPS, sem desconsiderar as características da territorialidade do serviço. Ainda, sugere estudos futuros baseados em Análises de *Clusters*, para analisar os grupos de municípios embasados em suas características comuns. 

## Conclusão

Evidenciou-se uma correlação entre os procedimentos realizados e registrados no CAPS e os indicadores socioeconômicos, indicando multifatorialidade na oferta de cuidados às pessoas com TM. A relação estreita entre a oferta e os indicadores relacionados à renda demonstra que o registro do procedimento segue o crescimento do indicador. Nesse aspecto, o processo de trabalho do CAPS deve ser pensado vinculado à assistência holística, observando as condições de vida no território. 

Portanto, urge a necessidade de estratégias que minimizem as inconsistências nos registros, como auditorias periódicas para monitorar a qualidade das informações, estabelecimento de protocolos operacionais que orientem um fluxo de registro e a capacitação contínua dos profissionais por meio de Educação Permanente em Saúde, para que os dados sejam fidedignos às atividades realizadas e garantam ferramentas para subsidiar políticas públicas.

## Data Availability

Todos os dados gerados ou analisados durante este estudo estão incluídos neste artigo publicado.

# Translation: Relación entre la oferta de procedimientos de los centros de atención psicosocial y factores socioeconómicos: un estudio ecológico^*^

**DOI:** 10.1590/1518-8345.8045.4978

**Authors:** Anna Helen Silva Lima, Ardigleusa Alves Coelho, Gabriela Maria Cavalcanti Costa, Ricardo Alves de Olinda, Valdecir Carneiro da Silva

**Affiliations:** 1 Universidade Estadual da Paraíba, Departamento de Enfermagem, Campina Grande, PB, Brasil.; 2 Becaria del Conselho Nacional de Desenvolvimento Científico e Tecnológico (CNPq), Brasil.; 3 Universidade Estadual da Paraíba, Departamento de Estatística, Campina Grande, PB, Brasil.

**Keywords:** Salud Mental, Centro de Atención Psicosocial, Servicios Comunitarios de Salud Mental, Acceso a la Atención Sanitaria, Sistemas de Información en Salud, Gestión de la Salud.

## Abstract

**Objetivo:**

analizar la correlación entre la oferta de procedimientos realizados en los Centros de Atención Psicosocial y los indicadores socioeconómicos en municipios con poblaciones superiores a 100.000 habitantes.

**Método:**

este es un estudio ecológico que abarca 312 municipios y 1.127 servicios. En la modelización, se extrajeron datos de la serie histórica de producción ambulatoria del Sistema de Información en Salud y de indicadores socioeconómicos del Atlas del Desarrollo Humano.

**Resultados:**

solo 12 capitales registraron acciones de reducción de daños; El 93% de los registros son más frecuentes en las capitales. Se observó una correlación positiva entre la asistencia domiciliaria y el ingreso per cápita; y una correlación negativa entre la rehabilitación psicosocial y la tasa de analfabetismo. Cabe destacar que el Índice de Desarrollo Humano Municipal (IDHM) y la renta per cápita se correlacionaron con la mayoría de los procedimientos, mientras que la tasa de analfabetismo coincidió con el 64,7% de ellos. Se encontraron diferencias estadísticamente significativas entre municipios de tamaño mediano y grande.

**Conclusión:**

la relación entre los procedimientos registrados en los Centros de Atención Psicosocial y los indicadores socioeconómicos puede indicar la influencia de cuestiones multifactoriales en la atención en salud mental.

## Destacados

**(1)** La atención a la Salud Mental en Brasil está influida por las desigualdades sociales.

**(2)** La atención en los Centros Comunitarios de Salud Mental se concentra en centros grandes.

**(3)** Falta de notificación de los registros de procedimientos en los Sistemas de Información.

**(4)** Los procedimientos de los CAPS están principalmente correlacionados con el IDHM y los ingresos.

**(5)** La oferta de procedimientos de los CAPS está relacionada con factores socioeconómicos.

## Introducción

Los trastornos mentales (TM) son un problema global de salud pública y se encuentran entre las principales causas no fatales de muerte prematura, contribuyendo a la reducción de la expectativa de vida en unos 20 años en comparación con la población general^1^. Sin embargo, solo el 12% de las personas afectadas tiene acceso a servicios de salud en países de bajos ingresos, según el Relatório Mundial sobre Saúde Mental^2^ ^-^ ^3^. En Brasil, la financiación para la salud mental es insuficiente para cubrir las necesidades de atención, representando solo el 1,6% de los recursos totales en 2016^3^. El fortalecimiento de los servicios comunitarios de salud mental está previsto en el Plano de Ação Integral de Saúde Mental (2013 - 2030), que propone aumentar las instalaciones en el 80% de los países como estrategia para reducir las tasas de rehospitalización psiquiátrica^2^ ^-^ ^5^.

La reforma psiquiátrica brasileña reestructuró la atención de la salud mental, abarcando desde la regulación de los derechos hasta la oferta de una atención de base comunitaria^3^. En este contexto, los Centros de Atención Psicosocial (CAPS), regulados por las Ordenanzas n.º 224/1992^6^ y n.º 336/2002^7^ del Ministerio de Salud, constituyen servicios de referencia territorial, de carácter abierto, destinados a promover la autonomía y la (re)integración social de los usuarios^8^. Entre 1998 y 2020, el número de CAPS aumentó de 148 a 2.657 CAPS^9^, consolidándose como componentes de la Acción Estratégica de la Red de Atención Psicosocial (RAPS) y como dispositivos sustitutos del modelo de asilo, asegurando el tránsito de usuarios a través de servicios de diversas complejidades^1^ ^,^ ^10^. Sin embargo, a partir de 2016, el panorama político nacional comenzó a afectar negativamente la operativización de la CAPS, debido a la adopción de estrategias neoliberales y conservadoras que redujeron la financiación y desestructuraron la Política Nacional de Salud Mental (PNSM) mediante la difusión de documentos normativos y la publicación de notas técnicas^4^ ^,^ ^11^.

Las actividades realizadas en los CAPS se registran según la estandarización de procedimientos basada en la Tabla del Sistema de Gestión de Procedimientos, Medicamentos, Ortesis, Prótesis y Materiales Especiales del Sistema Unificado de Salud (SIGTAP), registrada utilizando instrumentos como el Registro de Acciones Ambulatorias de Salud - Psicosociales (RAAS - Psicosocial), el Boletín Consolidado de Producción Ambulatoria (BPA-C), y el Boletín Individualizado de Producción Ambulatoria (BPA-I). agrupados en los Sistemas de Información en Salud)^12^. La información proporcionada no se utiliza para fines de financiación, ya que la transferencia financiera de los CAPS es fija, dependiendo de su tipología^13^.

Sin embargo, aún no existe consenso sobre los objetos de evaluación de los servicios de salud mental en Brasil, debido a la necesidad de contemplar las subjetividades que implican la producción de atención^14^. En los CAPS, los instrumentos de evaluación utilizados con frecuencia se centran en la satisfacción del usuario y la percepción de los profesionales a través de escalas - Evaluación de la Satisfacción del Paciente con los Servicios de Salud Mental (SATIS-BR) y Evaluación del Impacto del Trabajo en Servicios de Salud Mental (Impacto-BR)^15^. Así, la evaluación y el seguimiento del servicio actúan como una herramienta crucial para garantizar servicios efectivos y eficientes, mediante la cualificación del acceso y la atención, además de garantizar que los escenarios de negligencia previamente presenciados no se repitan^16^ ^-^ ^17^.

A pesar de la influencia de las condiciones socioeconómicas en la enfermedad y el seguimiento de la atención^18^, el análisis de la relación entre los procedimientos realizados en los CAPS y los indicadores socioeconómicos municipales sigue siendo incipiente en el escenario científico. La evaluación de datos secundarios, utilizados históricamente principalmente para fines administrativos de organización de equipos, permite una comprensión más completa de las desigualdades que impregnan la prestación de atención, ya que facilita la identificación de patrones de atención en diferentes contextos territoriales. En el contexto de la salud mental, los datos de salud permiten el seguimiento y evaluación de servicios y políticas públicas, considerando las especificidades socioeconómicas regionales^13^.

Por ello, teniendo en cuenta la población establecida para la instalación de las diferentes modalidades del CAPS, el estudio optó por evaluar municipios medianos y grandes, que son los principales responsables de los desplazamientos en busca de servicios de salud^19^. Dicho esto, el objetivo es analizar la correlación entre la oferta de procedimientos realizados en los CAPS y los indicadores socioeconómicos en municipios con una población superior a 100.000 habitantes, de modo que, de este modo, se puedan derivar inferencias sobre la equidad en el acceso a los servicios de atención comunitaria para personas con trastornos mentales y conductuales en Brasil.

## Método

### Tipo de estudio

Se trata de un diseño de estudio ecológico, basado en el análisis de datos secundarios disponibles en el *Sistema de Informação Ambulatorial do Departamento de Informática do Sistema Único de Saúde* (SIA/SUS) (SIA/SUS^)(^ ^20^ y en el Atlas de Desarrollo Humano en Brasil, del *Programa das Nações Unidas para o Desenvolvimento* (PNUD)^21^. El estudio se llevó a cabo de acuerdo con las recomendaciones del *Strengthening the Reporting of Observational Studies in Epidemiology* (STROBE).

### Escenario de la investigación

La investigación abarcó 312 municipios brasileños, seleccionados en función de la estratificación de la población, que superaban los 100.000 habitantes, según datos del Censo de 2022^22^. Incluyendo las 26 capitales, el Distrito Federal y los demás municipios, distribuidos en las cinco regiones: 27 municipios en la Región Norte, 63 en el Noreste, 143 en el Sureste, 55 en el Sur y 24 en el Centro-Oeste. La selección de municipios basada en la estratificación poblacional se debió a una importante brecha de datos. En este contexto, se analizó la producción ambulatoria de un porcentaje de 1.127 CAPS (40%), independientemente de su tipología, de un total de 2.836 (100%) existentes en el país^22^. La distribución geográfica de los CAPS incluidos en la muestra es la siguiente: 53 ubicados en la Región Norte, 198 en el Noreste, 596 en el Sureste, 197 en el Sur y 83 en el Centro-Oeste.

### Población y muestra 

La muestra consistió en 23 procedimientos registrados en tres instrumentos utilizados en las rutinas de atención de los CAPS, a saber: RAAS - Psicosocial, BPA-C y BPA-I. Considerando 15 procedimientos registrados en el RAAS - Psicosocial: recepción nocturna de pacientes en CAPS; recepción de pacientes en el tercer turno en CAPS; recepción diurna de pacientes en CAPS; atención individual al paciente en CAPS; atención grupal de pacientes en CAPS; cuidado familiar en CAPS; atención domiciliaria para pacientes con CAPS; prácticas corporales en CAPS; prácticas expresivas y comunicativas en CAPS; atención a situaciones de crisis; acciones de rehabilitación psicosocial; promoción de la contractualidad; Seguimiento de adultos con sufrimiento o trastornos mentales derivados del consumo de crack, alcohol y otras drogas - Unidad de Refugio para Adultos y Seguimiento de la población infantil y adolescente con sufrimiento o trastornos mentales derivados del consumo de crack, alcohol y otras drogas - Unidad de Refugio para Niños y Adolescentes. Mientras que en el BPA-C se registran un total de siete procedimientos, de los cuales: acciones para articular redes intra e intersectoriales; fortalecer el protagonismo de los usuarios de CAPS y sus familias; apoyo matricial a los equipos de atención primaria; acciones de reducción de daños; seguimiento del servicio residencial terapéutico por parte de CAPS; apoyo a servicios residenciales de naturaleza transitoria por parte de CAPS; Apoyo matricial de equipos de puntos de atención urgente y de urgencias y servicios hospitalarios de referencia para la atención de personas con sufrimiento o trastornos mentales y con necesidades de salud derivadas del consumo de alcohol, crack y otras drogas. Finalmente, la recepción inicial por parte de CAPS, registrada en el BPA-I.

El criterio de inclusión fue la existencia de registros presentes en al menos el 10% de los 312 municipios analizados, eliminándose cinco procedimientos: Acciones de articulación de redes intra e intersectoriales; fortalecer el protagonismo de los usuarios de CAPS y sus familias; Apoyo matricial para equipos de atención primaria; Seguimiento de la población infantil y adolescente con sufrimiento o trastornos mentales derivados del uso de crack, alcohol y otras drogas - Unidad de Recepción Infantil y Adolescente (UAI) y apoyo matricial de equipos de puntos de atención urgente y de emergencia y servicios hospitalarios de referencia para la atención de personas con sufrimiento o trastornos mentales y con necesidades de salud derivadas del consumo de alcohol, crack y otras drogas. La muestra final consistió en 18 procedimientos. Para los municipios, el criterio de inclusión observado fue la existencia de registros de procedimientos en al menos un año de la serie histórica y una población superior a 100 mil habitantes.

### Procedimientos e instrumentos para la recopilación de datos

La recopilación de datos se llevó a cabo durante el periodo de noviembre de 2023 a agosto de 2024, en dos etapas: (1) a través del Sistema de Información Ambulatoria del Departamento de Informática del Sistema Unificado de Salud^20^ (https://datasus.saude.gov.br); y (2) a través del Atlas del Desarrollo Humano en Brasil^21^ (https://www.atlasbrasil.org.br). 

En la etapa 1, inicialmente, se recopilaron datos secundarios obtenidos por la SIA/SUS en los registros de procedimientos (variables dependientes) en CAPS autorizados por el Ministerio de Salud que presentaban producción ambulatoria utilizando los instrumentos RAAS, BPA-C y BPA-I.

Para la etapa 2, las variables independientes fueron los indicadores socioeconómicos disponibles en el Atlas de Desarrollo Humano en Brasil: Índice de Desarrollo Humano Municipal (IDHM), índice Gini, índice Theil L, tasa de desempleo (población de 18 años o más), tasa de analfabetismo (población de 15 años o más), ingreso per cápita (salario mínimo) y proporción de personas pobres.

### Procedimientos de análisis de datos

Inicialmente, se realizó un análisis descriptivo de los datos con cálculos y comparaciones de la oferta media en los procedimientos en los CAPS, considerando grupos de municipios con poblaciones entre 100.000 y 199.999 habitantes (tamaño medio) y aquellos con más de 200.000 habitantes (tamaño grande). Para las variables dependientes, la media aritmética simple de la serie histórica (2016 a 2022) fue calculada por el municipio. Los datos sobre la oferta de procedimientos se estandarizaron para comparar entre los grupos de municipios según el número de poblaciones, calculando el número medio de procedimientos en la serie histórica por el número de habitantes y multiplicando el resultado por 10.000 habitantes.

Después de eso, se utilizó el *software* libre Jamovi, versión 2.3^23^, para aplicar las pruebas estadísticas. Primero, se realizó la prueba de Shapiro-Wilk, que indica que los datos no son paramétricos. La prueba de correlación estadística de Spearman se realizó para evaluar la oferta de procedimientos realizados en el CAPS y los indicadores socioeconómicos. Además, se realizó la ANOVA no paramétrica de Kruskal-Wallis para comparar la oferta de procedimientos entre los grupos de municipios según su tamaño. La significación estadística se estableció en un 5%, es decir, un valor de p<0,05.

### Aspectos éticos

Según la Resolución del Consejo Nacional de Salud n.º 510/2012^24^, la presentación de investigaciones queda exenta cuando se utilizan datos de dominio público y acceso abierto.

## Resultados

La distribución de los procedimientos ofertados por los municipios fue la siguiente: asistencia individual (100%), asistencia familiar (99,6%), asistencia grupal (99,3%), acogida diurna (99%), acogida inicial (98,7%), asistencia domiciliaria (97,7%), atención en situaciones de crisis (94,5%), prácticas expresivas y comunicativas (94,2%), prácticas corporales (93,2%), acciones de rehabilitación psicosocial (92,9%), promoción de la contractualidad (86,8%), acogida nocturna (68,9%), acogida en el tercer turno (53,8%), seguimiento del paciente en el Servicio Residencial Terapéutico (28,2%), seguimiento en el Servicio Residencial Terapéutico (20,1%), acciones de reducción de daños (15%) y seguimiento de adultos en una Unidad de Acogida para Adultos (10%).

La Tabla 1 muestra la tasa (por cada 10 mil habitantes) de los procedimientos en los CAPS según la producción ambulatoria en las capitales brasileñas. Destacan registros de procedimientos con altas tasas para las capitales de las siguientes regiones: Centro-Oeste (Brasilia y Campo Grande); Norte (Palmas y Río Branco); Noreste (João Pessoa y Recife); Sureste (São Paulo y Vitória); y Sur (Curitiba y Porto Alegre).

**Tabla 1:** Tasas (por 10 mil habitantes) de los procedimientos en los CAPS* en capitales brasileñas, 2016 a 2022. Brasil, 2024

Región	Capitales	Procedimientos							
		Acogida nocturna	Acogida en el tercer turno	Seguimiento de paciente en SRT^†^	Acogida diurna	Asist. individual	Asist. en grupo	Asistencia. familiar	Acogida inicial
Centro- Oeste	Brasília	1,02	0,43	-	18,21	102,2	33,72	16,13	16,62
	Campo Grande	68,24	0,03	1,44	76,82	508,44	142,78	74,34	27,98
	Cuiabá	-	-	-	4,79	95,78	55,46	39,55	20,62
	Goiânia	0,12	0,10	1,58	2,54	63,21	29,33	13,53	7,14
Norte	Boa Vista	6,95	16,12	-	-	61,70	39,31	5,65	25,52
	Belém	1,68	-	-	5,00	186,25	17,56	53,64	19,32
	Macapá	-	-	-	6,21	47,80	28,82	10,34	-
	Manaus	1,12	-	-	3,33	123,94	11,69	35,70	12,85
	Palmas	14,25	1,12	-	56,19	555,70	182,20	70,57	62,88
	Rio Branco	12,76	-	-	20,47	230,34	10,72	3,30	9,99
Noreste	Aracaju	18,79	0,17	15,80	74,63	153,33	66,10	63,67	9,55
	Fortaleza	0,11	0,55	0,22	16,92	78,90	11,51	2,13	24,40
	João Pessoa	11,92	11,98	-	302,87	101,04	65,84	7,17	2,92
	Maceió	7,81	2,22	0,01	97,06	169,31	141,31	39,29	14,37
	Natal	10,88	-	0,96	3,53	138,00	119,50	10,50	10,05
	Recife	24,61	8,18	20,25	75,77	285,35	199,50	84,83	30,16
	Salvador	0,41	0,01	0,47	74,21	113,84	41,04	21,98	6,08
	São Luís	0,88	0,01	-	109,33	115,68	57,30	30,47	2,69
	Teresina	26,15	-	2,07	63,90	275,90	145,82	116,50	6,86
Sureste	Belo Horizonte	34,17	15,23	11,90	256,55	193,48	11,95	1,09	4,11
	Rio de Janeiro	7,37	6,19	4,01	45,55	120,99	24,61	28,76	8,83
	São Paulo	26,41	6,10	1,56	116,68	327,15	147,52	70,46	33,95
	Vitória	38,03	0,13	-	226,32	296,81	86,11	45,49	36,23
Sur	Curitiba	87,79	2,52	1,43	81,14	661,59	311,37	131,73	50,66
	Florianópolis	0,02	0,03	-	10,67	172,57	196,33	16,70	32,33
	Porto Alegre	17,33	8,04	-	32,33	224,33	45,57	33,83	18,54
**Región**	**Capitales**	**Procedimientos**								
		**Asistencia domici-liaria**	**Prácticas corporales**	**Prácticas expresivas**	**Atención a las situaciones de crisis**	**Acciones de reducción de daños**	Seguimiento de SRT^†^ por CAPS*	**Acciones de rehabilitación psicosocial**	**Promoción de la contractualidad**	**Seguimiento de personas adultas en UAA^‡^**
Centro- Oeste	Brasília	0,76	2,89	398	6,64	0,01	0,04	9,15	1,82	-
	Campo Grande	1,25	13,16	28,53	80,25	-	-	3,82	3,50	1,11
	Cuiabá	5,08	0,41	20,15	0,86	0,12	-	1,35	1,25	-
	Goiânia	0,53	5,10	3,80	0,92	0,02	0,03	1,98	1,79	-
Norte	Belém	1,33	10,05	9,20	3,02	-	0,21	9,20	11,23	-
	Boa Vista	1,03	5,84	1,17	2,44	0,10	0,46	-	0,05	0,86
	Macapá	0,05	0,05	2,22	3,57	-	-	0,07	0,04	-
	Manaus	0,88	6,69	6,12	2,01	-	0,14	6,12	7,47	-
	Palmas	18,75	13,06	5,15	2,63	0,36	0,45	13,89	0,51	-
	Porto Velho	4,64	32,51	8,41	7,13	0,10	-	3,43	0,34	-
	Rio Branco	0,56	0,03	0,82	-	0,59	-	6,13	7,68	4,62
Noreste	Aracaju	9,72	10,71	36,19	41,22	-	-	38,11	7,80	1,36
	Fortaleza	0,66	0,20	0,14	0,28	-	0,04	0,59	0,04	0,16
	João Pessoa	1,40	1,92	25,89	4,70	-	26,70	3,97	0,06	-
	Maceió	4,55	13,03	15,94	4,23	-	0,01	4,86	1,00	-
	Natal	0,14	0,95	2,76	2,07	-	0,01	0,79	0,24	-
	Recife	11,13	6,13	17,35	22,20	0,12	0,01	8,74	26,45	1,85
	Salvador	2,09	10,81	30,78	1,89	-	-	6,42	4,32	-
	São Luís	11,61	20,13	25,54	37,17	-	-	19,02	0,30	-
	Teresina	7,78	10,45	35,61	58,18	-	-	7,68	-	-
Sureste	Belo Horizonte	12,18	4,46	3,61	5,75	0,01	-	0,88	5,02	-
	Rio de Janeiro	2,75	6,98	22,00	12,21	1,08	0,03	11,63	6,81	-
	São Paulo	14,65	29,84	85,10	14,00	-	-	49,28	24,52	0,38
	Vitória	3,12	3,38	112,34	8,49	-	-	19,45	10,83	-
Sur	Curitiba	4,75	28,03	44,43	68,98	-	0,02	80,13	36,37	-
	Florianópolis	0,06	4,47	16,26	5,53	-	-	3,23	6,27	-
	Porto Alegre	0,91	9,70	10,41	6,58	2,56	0,01	22,50	6,89	0,01

Fuente: SIA/SUS _^(20)^_ , 2016-2022.*CAPS = Centro de Atención Psicosocial; ^†^SRT = Servicio de Residencia Terapéutica; ^‡^UAA = Unidad de Acogida de Adultos

En este contexto, como se muestra en la Tabla 2, elaborada a partir de la oferta de procedimientos en capitales no medianas, cabe destacar que ciertas Unidades Federativas no tienen ocurrencias de municipios no capitales en la muestra, ya que los municipios del interior no tienen el tamaño poblacional establecido en la investigación.La Tabla 3 presenta el escenario de los no capitales con municipios grandes. Se observaron ocurrencias con tasas altas en las siguientes regiones que no son capitales de estado: Centro-Oeste (Mato Grosso y Mato Grosso do Sul); Norte (Rondônia y Pará); Noreste (Maranhão y Paraíba); Sureste (Minas Gerais y São Paulo); y Sur (Paraná y Santa Catarina).

Cabe destacar que procedimientos como la recepción en el turno de terceros y la atención nocturna se concentran en este grupo, ya que están restringidos a servicios que funcionan sin interrupciones, las 24 horas del día. Por lo tanto, la falta de registros de estos procedimientos en municipios de tamaño mediano que no son capitales es un hallazgo esperado en el conjunto de datos analizados.

**Tabla 2:** Tasas medias (por cada 10 mil habitantes) de los procedimientos CAPS* en municipios de tamaño mediano no capitales, 2016 a 2022. Brasil, 2024

Región	No capitales	Procedimientos							
		Acogida nocturna	Acogida en el tercer turno	Seguimiento de paciente en SRT^†^	Acogida diurna	Asistencia individual	Asistencia en grupo	Asistencia familiar	Acogida inicial
Centro-Oeste	Goiás (No capital)	0,16	0,07	1,65	15,21	129,03	67,45	6,01	18,32
	Mato Grosso (No capital)	0,09	-	-	61,83	194,70	26,89	52,07	26,00
	Mato Grosso do Sul (No capital)	-	-	-	50,62	151,46	0,30	6,88	19,81
Norte	Amapá (No capital)	-	-	-	1,17	17,79	5,70	0,75	1,63
	Amazonas (No capital)	3,23	-	-	3,94	162,54	67,71	20,14	13,63
	Pará (No capital)	6,27	1,24	-	47,99	227,16	112,74	65,39	18,16
	Rondônia (No capital)	0,38	-	-	58,78	138,38	2,32	0,14	0,37
Noreste	Bahia (Não capital)	0,45	0,50	-	154,89	267,26	120,32	83,49	29,63
	Ceará (No capital)	1,22	0,13	-	49,17	241,49	18,22	15,25	20,73
	Maranhão (No capital)	27,80	26,40	-	816,66	843,12	209,06	84,45	114,72
	Paraíba (No capital)	1,78	1,73	1,04	72,59	541,96	5678,58	718,45	22,35
	Pernambuco (No capital)	12,74	18,03	12,85	18,12	170,13	186,90	110,74	19,91
	Piauí (No capital)	-	0,98	-	0,22	28,47	4,31	0,39	0,10
	Rio Grande do Norte (No capital)	1,58	-	-	44,49	116,76	31,17	51,25	0,11
	Sergipe (No capital)	0,45	8,12	233,02	24,23	230,68	268,88	70,25	11,09
Sureste	Espírito Santo (No capital)	0,42	0,15	-	123,43	169,93	42,84	14,28	9,02
	Minas Gerais (No capital)	26,75	7,60	73,71	169,92	424,85	127,07	63,48	35,34
	Rio de Janeiro (No capital)	0,82	0,24	6,13	236,19	160,75	114,21	44,56	26,12
	São Paulo (No capital)	8,11	0,42	8,88	100,14	285,34	118,81	45,71	15,60
Sur	Paraná (No capital)	0,55	1,18	-	102,73	257,47	258,92	56,41	20,64
	Rio Grande do Sul (No capital)	6,62	0,47	2,55	32,79	338,96	105,49	56,34	28,92
	Santa Catarina (No capital)	0,81	27,28	-	52,52	309,46	178,26	49,80	35,74
**Región**	**No capitales**	**Procedimientos**								
		**Asistencia domi-ciliaria**	**Prácticas corporales**	**Prácticas expresivas**	**Atención a las situaciones de crisis**	**Acciones de reducción de daños**	Seguimiento de SRT^†^ por CAPS*	**Acciones de rehabilitación psicosocial**	**Promoción de la contractualidad**	**Seguimiento de personas adultas en UAA^‡^**
Centro-Oeste	Goiás (No capital)	7,07	10,56	21,94	1,85	2,53	0,20	1,30	1,42	-
	Mato Grosso (No capital)	7,65	14,15	37,48	5,55	2,31	0,21	30,98	13,83	-
	Mato Grosso do Sul (No capital)	1,85	-	39,09	4,82	-	-	0,90	1,32	-
Norte	Amapá (No capital)	-	4,01	4,74	3,15	-	-	9,62	2,03	-
	Amazonas (No capital)	2,22	0,95	7,15	5,91	1,20	-	15,21	0,51	-
	Pará (No capital)	3,13	42,92	47,54	7,97	-	-	24,67	3,20	-
	Rondônia (No capital)	10,44	-	-	-	-	-	17,48	2,70	-
Noreste	Maranhão (No capital)	1,93	30,87	22,60	47,81	-	0,60	84,58	0,09	-
	Bahia (No capital)	20,56	55,67	80,21	2,28	0,27	-	45,86	40,45	-
	Ceará (No capital)	6,14	1,53	0,11	2,06	-	0,08	0,28	-	-
	Paraíba (No capital)	7,77	706,94	25,23	3,13	0,10	-	2,13	0,99	-
	Pernambuco (No capital)	6,36	8,66	33,95	45,55	-	0,47	7,40	42,62	0,63
	Piauí (No capital)	0,09	0,06	-	-	-	-	0,31	0,06	-
	Rio Grande do Norte (No capital)	1,42	1,65	2,66	0,08	-	-	0,55	0,17	-
	Sergipe (No capital)	9,08	33,35	37,04	0,78	-	0,44	2,87	1,08	3,99
Sureste	Espírito Santo (No capital)	5,67	6,52	12,89	0,72	-	-	0,84	0,69	-
	Minas Gerais (No capital)	16,64	29,49	54,39	58,64	0,14	0,08	10,57	5,72	2,51
	Rio de Janeiro (Não capital)	7,90	11,24	29,71	6,91	0,08	6,20	16,52	7,42	-
	São Paulo (Não capital)	13,81	28,59	30,33	10,21	0,56	0,17	22,58	13,29	5,70
Sul	Paraná (No capital)	11,86	30,55	67,16	9,35	-	-	42,80	22,82	0,21
	Rio Grande do Sul (No capital)	19,35	11,52	24,39	7,83	6,99	0,10	11,08	10,09	-
	Santa Catarina (No capital)	8,64	11,70	43,46	3,97	1,13	-	28,55	5,47	0,71

Fuente: SIA/SUS^20^, 2016-2022. *CAPS = Centro de Atención Psicosocial; ^†^SRT = Servicio Residencial Terapéutico; ^‡^UAA = Unidad de Acogida de Adultos

**Tabla 3:** Tasas medias (por cada 10 mil habitantes) de los procedimientos CAPS* ofrecidos en municipios no capitales de gran porte, 2016 a 2022. Brasil, 2024

Región	No capitales	Procedimientos							
		Acogida nocturna	Acogida en tercer turno	Seguimiento de paciente en SRT^†^	Acogida diurna	Asistencia individual	Asistencia en grupo	Asistencia familiar	Acogida inicial
Centro-Oeste	Goiás (No capital)	8,12	0,18	-	18,14	97,10	85,92	29,38	20,78
	Mato Grosso (No capital)	0,73	0,51	-	4,81	175,15	472,58	52,22	27,99
	Mato Grosso do Sul (No capital)	1,88	0,04	-	10,46	71,42	4,75	8,24	5,31
Norte	Pará (No capital)	15,97	0,30	-	41,27	217,30	102,62	47,12	19,31
Noreste	Alagoas (No capital)	0,09	0,06	-	29,43	139,54	97,42	25,52	22,19
	Bahia (No capital)	3,58	0,11	-	30,48	129,22	51,47	38,62	22,65
	Ceará (No capital)	6,99	0,10	3,57	98,70	329,65	83,86	39,74	13,56
	Maranhão (No capital)	1,71	0,63	0,81	105,78	35,51	93,65	17,34	16,71
	Paraíba (Não capital)	0,17	0,06	3,50	12,92	483,69	292,65	127,90	17,50
	Pernambuco (No capital)	13,19	3,67	7,46	56,14	191,27	154,90	67,83	22,83
	Rio Grande do Norte (No capital)	14,74	-	-	81,82	185,53	231,96	26,58	14,18
Sureste	Espírito Santo (No capital)	0,68	0,04	-	30,85	60,50	23,54	9,82	6,88
	Minas Gerais (No capital)	19,61	1,86	10,39	164,55	294,96	103,72	56,44	20,94
	Rio de Janeiro (No capital)	4,06	0,81	3,10	44,27	136,23	83,19	27,55	9,19
	São Paulo (No capital)	23,56	4,03	7,09	91,52	487,80	171,91	61,98	24,01
Sur	Paraná (No capital)	9,36	4,82	3,79	34,05	260,29	240,47	78,52	21,88
	Rio Grande do Sul (No capital)	23,96	14,04	7,75	60,54	306,84	130,84	61,13	34,05
	Santa Catarina (No capital)	25,04	11,49	-	83,05	334,93	236,71	41,15	23,87
**Región**	**No capitales**	**Procedimientos**								
		**Asistencia domiciliaria**	**Prácticas corporales**	**Prácticas expresivas**	**Atención a las situaciones de crisis**	**Acciones de reducción de daños**	Seguimiento de SRT^†^ por CAPS*	**Acciones de rehabilitación psicosocial**	**Promoción de la contractualidad**	**Seguimiento de personas adultas en UAA^‡^**
Centro-Oeste	Goiás (No capital)	12,15	26,97	31,50	16,49	2,64	0,65	26,79	19,70	-
	Mato Grosso (No capital)	5,81	61,34	9,04	2,95	-	-	5,84	0,65	-
	Mato Grosso do Sul (No capital)	6,68	1,17	4,55	0,49	4,73	-	4,14	0,42	-
Norte	Pará (No capital)	5,04	45,22	22,77	4,80	0,22	-	3,12	1,77	-
Noreste	Maranhão (No capital)	1,17	10,84	32,30	5,62	-	0,14	59,78	5,10	-
	Alagoas (No capital)	2,50	1,73	2,32	4,82	-	0,43	0,19	0,85	-
	Bahia (No capital)	2,58	5,21	7,74	6,48	23,48	0,53	10,37	3,85	-
	Ceará (No capital)	6,08	18,43	17,58	20,08	3,08	2,12	1,27	8,69	6,22
	Paraíba (No capital)	11,28	22,20	82,76	2,27	-	-	10,93	2,59	-
	Pernambuco (No capital)	3,67	10,14	26,38	33,07	-	0,02	8,43	7,44	0,11
	Rio Grande do Norte (No capital)	1,18	11,39	1,30	0,14	-	-	0,16	-	-
Sureste	Espírito Santo (No capital)	0,49	3,74	12,94	3,20	-	-	2,60	1,57	-
	Minas Gerais (No capital)	6,76	19,43	40,10	21,67	0,04	0,04	15,33	11,85	0,61
	São Paulo (No capital)	10,31	23,94	34,89	28,70	0,18	0,06	34,48	34,37	0,61
	Rio de Janeiro (No capital)	2,04	19,33	38,57	3,38	3,03	0,32	10,82	3,67	-
Sur	Paraná (No capital)	14,21	16,82	64,14	6,96	-	-	22,43	17,17	3,76
	Rio Grande do Sul (No capital)	7,66	21,74	40,73	10,90	4,33	0,08	16,81	7,79	2,34
	Santa Catarina (No capital)	13,79	14,14	49,14	10,36	3,58	-	25,11	23,68	-

Fuente: SIA/SUS^20^, 2016 - 2022. *CAPS= Centro de Atención Psicosocial; ^†^SRT= Servicio Residencial Terapéutico; ^‡^UAA= Unidad de Acogida de Adulto

Además, la comparación entre los registros de los procedimientos ofrecidos en CAPS en las capitales y no capitales muestra que las medias más altas de registros corresponden a la recepción diurna, atención individual y atención grupal (Tabla 4).

**Tabla 4:** Comparación entre las tasas medias (por cada 10 mil habitantes) de la oferta de procedimientos en capitales y no capitales de CAPS*, con una población superior a 100 mil habitantes, 2016 a 2022. Brasil, 2024

Procedimientos realizados en CAPS*	Media de registros de la serie histórica (2016 a 2022)	
	Capitales (n^†^=26)	No capitales (n^†^=286)
Acogida nocturna de paciente en CAPS*	16,82	12,68
Acogida en tercer turno de paciente en CAPS*	3,96	4,29
Seguimiento de paciente en SRT^†^	4,75	18,18
Acogida diurna de paciente en CAPS*	68,76	106,28
Asistencia individual de paciente en CAPS*	204,48	296,77
Asistencia en grupo de paciente en CAPS*	82,62	176,49
Asistencia familiar en CAPS*	39,19	56,53
Acogida inicial por CAPS*	19,03	23,69
Asistencia domiciliaria para pacientes de CAPS*	4,55	9,70
Prácticas corporales en CAPS*	9,22	28,41
Prácticas expresivas y comunicativas en CAPS*	21,26	36,68
Atención a las situaciones de crisis	15,51	16,25
Acciones de reducción de daños	0,39	6,39
Seguimiento de SRT^‡^ por CAPS*	1,76	0,83
Acciones de rehabilitación psicosocial	12,78	21,6
Promoción de contractualidad	6,64	15,07
Seguimiento de personas adultas con sufrimiento o trastornos mentales derivados del uso de *crack*, alcohol y otras drogas - UAA^§^	1,15	1,74

Fuente: SIA/SUS^20^, 2016-2022. *CAPS = Centro de Atención Psicosocial; ^†^n = Número de participantes; ^‡^SRT = Servicio Residencial Terapéutico; ^§^UAA = Unidad de Acogida de Adultos

Se observó una correlación significativa entre los indicadores socioeconómicos y la provisión de los siguientes procedimientos: recepción diurna; atención grupal; cuidado familiar; atención domiciliaria; prácticas corporales; prácticas expresivas y comunicativas; atención en situaciones de crisis; y la promoción de la contractualidad (Tabla 5).

**Tabla 5:** Análisis de la correlación entre la provisión de procedimientos en Centros de Atención Psicosocial y los indicadores socioeconómicos de municipios medianos y de gran porte en Brasil, 2016 a 2022

	Análisis de la correlación	Índice de Theil-L	Índice de Gini	Taxa de desocupación	IDHM*	Tasa de analfabetismo	Renta per cápita	Proporción de pobres
Acogida nocturna de paciente en CAPS^†^	*rs* ^*‡*^	0,204	0,209	-0,058	0,191	-0,075	0,191	-0,079
	*Valor de p* ^*§*^	0,003	0,002	0,401	0,005	0,274	0,005	0,246
Acogida diurna de paciente en CAPS^†^	*rs* ^*‡*^	0,019	0,028	-0,017	0,205	-0,137	0,167	-0,141
	*Valor de p* ^*§*^	0,740	0,626	0,767	<0,001	0,016	0,003	0,013
Asistencia individual de paciente en CAPS ^†^	*rs* ^*‡*^	0,035	0,043	-0,167	0,165	-0,116	0,148	-0,144
	*Valor de p* ^*§*^	0,535	0.452	0,003	0,004	0,041	0,009	0,011
Asistencia en grupo de paciente en CAPS^†^	*rs* ^*‡*^	-0,138	0,127	-0,136	0,200	-0,178	0,171	-0,230
	*Valor de p* ^*§*^	0,015	0,025	0,017	<0,001	0,002	0,003	<0,001
Asistencia familiar^†^	*rs* ^*‡*^	-0,008	0,003	-0,067	0,168	-0,154	0,161	-0,158
	*Valor de p* ^*§*^	0,889	0,960	0,239	0,003	0,007	0,005	0,005
Asistencia domiciliaria para pacientes^†^	*rs* ^*‡*^	-0,095	-0,076	-0,267	-0,166	0,235	0,296	-0,288
	*Valor de p* ^*§*^	0,098	0,184	<0,001	<0,001	<0,001	<0,001	<0,001
Prácticas expresivas y comunicativas	*rs* ^*‡*^	-0,052	-0,043	-0,130	0,144	-0,155	0,172	-0,219
	*Valor de p* ^*§*^	0,371	0,467	0,026	0,014	0,008	0,003	<,001
Atención a las situaciones de crisis	*rs* ^*‡*^	0,090	0,104	-0,046	0,194	-0,129	0,171	-0,132
	*Valor de p* ^*§*^	0,123	0,074	0.433	<,001	0,027	0,003	0,023
Acciones de rehabilitación psicosocial	*rs* ^*‡*^	0,023	0,038	-0,170	0,285	-0,238	0,288	-0,271
	*Valor de p* ^*§*^	0,700	0,520	0,004	<,001	<,001	<,001	<,001
Promoción de contractualidad	*rs* ^*‡*^	0,070	0,082	-0,126	0,210	-0,189	0,238	-0,232
	*Valor de p* ^*§*^	0,251	0,002	0,038	<0,001	0,002	<0,001	<0,001

Fuente: SIA/SUS^20^,2016-2022; PNUD^21^, 2010; *The Jamovi Project* ^23^. *IDHM = Índice de Desarrollo Humano Municipal; ^†^CAPS = Centro de Atención Psicosocial; ^‡^ *rs =* Coeficiente de correlación de Spearman; ^§^Valor de p = Probabilidad de significación

En cuanto a las correlaciones, se demuestra que el rendimiento de los procedimientos mencionados está influido por los indicadores analizados presentados con valores positivos, es decir, los registros de procedimientos aumentan con el indicador. Además, hubo una correlación negativa, presentada mediante una relación inversamente proporcional. 

El análisis utilizando la prueba de Kruskal-Wallis (ANOVA no paramétrica) mostró diferencias estadísticamente significativas entre municipios medianos y de gran porte para los procedimientos: acogida nocturna (χ²=7,26; p = 0,007), asistencia en grupo (χ²=4,27; p = 0,039), asistencia familiar (χ² = 7,07; p = 0,008), atención en situación de crisis (χ²=7,20; p = 0,007) y seguimiento de SRT por CAPS (χ² = 12,10; p= < 0,001).

Las comparaciones múltiples mostraron significación estadística, indicando que los municipios grandes tienen tasas más bajas en comparación con los municipios medianos para los procedimientos: cuidado nocturno (W = - 3,81; p = 0,007), cuidado grupal (W = - 2,92, p = 0,039), cuidado familiar (W = -2,92; p = 0,008) y cuidado en situaciones de crisis (W = -3,79; p = 0,007). Mientras que el procedimiento de seguimiento SRT mostró valores significativamente más altos en municipios de gran porte (W = 4,92; p = <0,001).

## Discusión

Los hallazgos indican la relación entre los factores socioeconómicos y los procedimientos realizados en los servicios brasileños de base comunitaria, considerando la influencia de las disparidades sociales y económicas en el acceso y la garantía de atención en los servicios^25^. Aunque las correlaciones observadas muestran una magnitud baja, apuntan al crecimiento de registros en municipios con mejores indicadores socioeconómicos, como aquellos con valores más altos de IDHM y renta per cápita, que presentaron correlaciones positivas.

Por otro lado, se identificaron correlaciones negativas, especialmente con el indicador de tasa de analfabetismo, evidenciado por el aumento de los registros en detrimento de los valores más bajos del indicador. En este contexto, otras producciones^26^ ^-^ ^27^ han demostrado que las personas con menos educación tienen un bajo acceso a la atención primaria, lo que provoca una búsqueda tardía de servicios de salud. Así, la ausencia de alfabetización en salud, influenciada por varios factores, incluido el escolarizado, dificulta el acceso y la continuidad de la atención^28^.

Al observar las tasas de provisión de procedimientos, se identifica un patrón nacional en los registros, de modo que los procedimientos de reducción de daños, el seguimiento SRT y el seguimiento de personas en la UAA se redujeron tanto en capitales como en no capitales medianos y de gran porte. Cabe destacar que, entre estos procedimientos, solo el seguimiento de personas en UAA mostró una correlación estadísticamente significativa con indicadores socioeconómicos; sin embargo, la reducción del registro puede limitar la precisión del análisis.

El registro limitado de procedimientos relacionados con servicios que apoyan la atención en salud mental, como la SRT y la UAA, está relacionado con la gestión de la atención de mayor complejidad, observando las características organizativas. Sin embargo, se observa que la atención proporcionada a los residentes en las SRT se realiza de manera unidireccional, con solo el desplazamiento de usuarios al CAPS de referencia, ya que el seguimiento de las personas que viven en los SRT tiene registros altos, mientras que hay poco registro de las actividades realizadas en los SRT. Se enfatiza que la relación entre los servicios debe ser estrecha, ya que el CAPS actúa como servicio de referencia para residencias terapéuticas^29^.

La ausencia de registros para los procedimientos relacionados con la comunicación del CAPS con otros dispositivos de red demuestra la limitación de la atención proporcionada a la red especializada en salud mental, lo que puede resultar en la restricción del tránsito de usuarios a través de los diferentes niveles de atención y en su cronicidad en el servicio^15^. Además, la falta de un sistema unificado de referencia y contrarreferencia que comunique la Atención Primaria de Salud, combinada con el número limitado de equipos de APS formados para atender las demandas de salud mental, sobrecarga el CAPS y expone barreras estructurales y humanas^30^ ^-^ ^31^.

La diferencia significativa en la oferta de procedimientos según el tamaño de la población, que indica municipios más grandes con tasas más bajas, puede deberse a la mayor capilaridad de la red en municipios más grandes, cuya oferta de salud mental puede realizarse en otros servicios debido a la existencia de una red más consolidada^32^. Además, existe una tendencia hacia la internalización de la atención en salud mental en ciudades medianas y pequeñas, principalmente con la participación de la APS^33^. Este punto queda demostrado por la evolución del indicador de cobertura CAPS/100 mil habitantes presentado por el informe Salud Mental en Datos^34^, que menciona varios municipios del interior con cobertura CAPS superior al 50%.

Los registros de los procedimientos presentan carencias significativas, lo que contribuye al debilitamiento de los datos y a la falta de conocimiento concreto sobre la oferta de procedimientos en los servicios de salud mental. En este contexto, la acción de registrar suele estar relacionada únicamente con la actividad burocrática del proceso de trabajo^35^. Además, los profesionales consideran que las dificultades relacionadas con el registro se minimizarían si se reforzara el proceso de formación; por lo tanto, la relevancia de las cualificaciones unificadas y periódicas está alineada con las políticas de salud^36^. Cabe destacar que la consolidación de la cultura de registrar, monitorizar y evaluar indicadores de salud mental califica la producción de evidencia para apoyar decisiones coherentes con las necesidades de la población^37^.

La limitación del estudio está vinculada a las limitaciones inherentes al diseño de la investigación y al uso de datos secundarios. Se destaca la incompletitud de los datos puestos a disposición por los servicios de los municipios en la muestra en los Sistemas de Información, lo que hizo imposible realizar un análisis más específico por municipio y requirió la agregación, mediante media aritmética, de la serie histórica. Además, los datos se generan de forma acumulativa por el municipio, y no es posible identificar ni diferenciar el tipo de CAPS que registró el procedimiento. En este sentido, la ausencia de registros dificulta establecer la planificación estratégica de la Red de Atención Psicosocial y conocer sus características.

Sin embargo, es importante destacar que esta investigación buscó contribuir al debate discutiendo la provisión de procedimientos desde una perspectiva socioeconómica, observando la multifactorialidad que afecta al proceso de trabajo en el CAPS, sin despreciar las características territoriales del servicio. También sugiere futuros estudios basados en el Análisis de Clústeres, para analizar los grupos de municipios según sus características comunes.

## Conclusión

Se evidenció una correlación entre los procedimientos realizados y registrados en el CAPS y los indicadores socioeconómicos, lo que indica multifactorialidad en la prestación de atención a personas con TM. La estrecha relación entre los indicadores relacionados con la oferta y los ingresos demuestra que el registro del procedimiento sigue al crecimiento del indicador. En este aspecto, el proceso de trabajo del CAPS debe considerarse vinculado a la atención holística, observando las condiciones de vida en el territorio.

Por ello, existe una necesidad urgente de estrategias que minimicen las inconsistencias en los registros, como auditorías periódicas para monitorizar la calidad de la información, el establecimiento de protocolos operativos que guíen el flujo de registro y la formación continua de profesionales a través de la Educación Permanente en Salud, para que los datos sean fiables para las actividades realizadas y garanticen herramientas que respalden las políticas públicas.

## Data Availability

Todos los datos generados o analizados durante este estudio están incluidos en este artículo publicado.
